# On characterization of entropy measure using logarithmic regression model for Copper(II) Fluoride

**DOI:** 10.1371/journal.pone.0300757

**Published:** 2024-03-26

**Authors:** Muhammad Kamran Siddiqui, Mazhar Hussain, Sana Javed, Sadia Khalid, Tayyaba Noor, Fikadu Tesgera Tolasa

**Affiliations:** 1 Department of Mathematics, COMSATS University Islamabad, Lahore Campus, Pakistan; 2 Department of Mathematics, Dambi Dollo University, Oromia, Ethiopia; Nazarbayev University, KAZAKHSTAN

## Abstract

The versatile uses of Copper(II) Fluoride (*CuF*_2_) are well known; these include its usage as a precursor in chemical synthesis as well as its contribution to the creation of sophisticated materials and electronics. There are interesting opportunities to study the interactions between these elements because of their unique crystal structure, which contains copper ions and fluoride anions. Its potential in optoelectronic devices and conductive qualities also make it a viable material for next-generation technologies. To better understand the structural properties of *CuF*_2_ and how they affect its entropy, we present new Zagreb indices in this study and use them to calculate entropy measures. We also build a regression model to clarify the relationship between the calculated indices and entropy levels. The findings of our investigation offer significant understanding regarding the ability of the suggested Zagreb indices to extract meaningful content and their correlation with entropy in the context of *CuF*_2_. This information is important for understanding *CuF*_2_ alloys and for exploring related complex materials.

## 1 Introduction

Graph theory is a branch of mathematics that deals with the study of graphs, which are mathematical structures used to model pairwise relations between objects. Within the realm of graph theory, a graph is a structure made up of nodes, also called vertices, and edges, which are the connecting lines. The number of vertices incident with a vertex *u* is called its degree and is denoted by Λ_*u*_ [1]. A graph’s size can be determined by counting its edges, and its order can be determined by counting its vertices. These basic ideas serve as the foundation for the analytical framework that is utilized to comprehend and investigate the wide range of characteristics and behaviors that graphs display. Graph theory offers a powerful toolkit for analyzing intricate network topologies and relationships with these fundamental ideas [2].

Topological indices are quantitative tools that provide a way to describe the complex topological structures that are intrinsic to each graph [3]. The principal benefit of utilizing these topological indices is their ability to reveal a wide range of characteristics related to molecules, chemical compounds, and networks without requiring a thorough understanding of their structural details. These indices are highly significant, particularly in the fields of drug design, QSAR studies, and chemical substance property prediction [4]. They simplify the candidate screening and optimization process, which makes them invaluable in both academic and professional settings. Beyond graph theory, topological indices are extremely useful for exploring and analyzing complicated systems and networks in an effective manner.

The topological index is a mathematical function: Top:F→R, where R represents the set of real numbers and F represents a simple graph. This fundamental concepts asserts that $Top(F_{1})$ is identical to $Top(F_{2})$ if two graphs, $F_{1}$ and $F_{2}$, are isomorphic [5]. These topological indices cover a broad spectrum of numerical forms that capture the structural information of a graph, such as polynomials, matrices, relational tables, and other numerical representations [6, 7]. Nadeem et al. [8] discussed the topological aspects of metal-organic structures.

**Fig 1:**
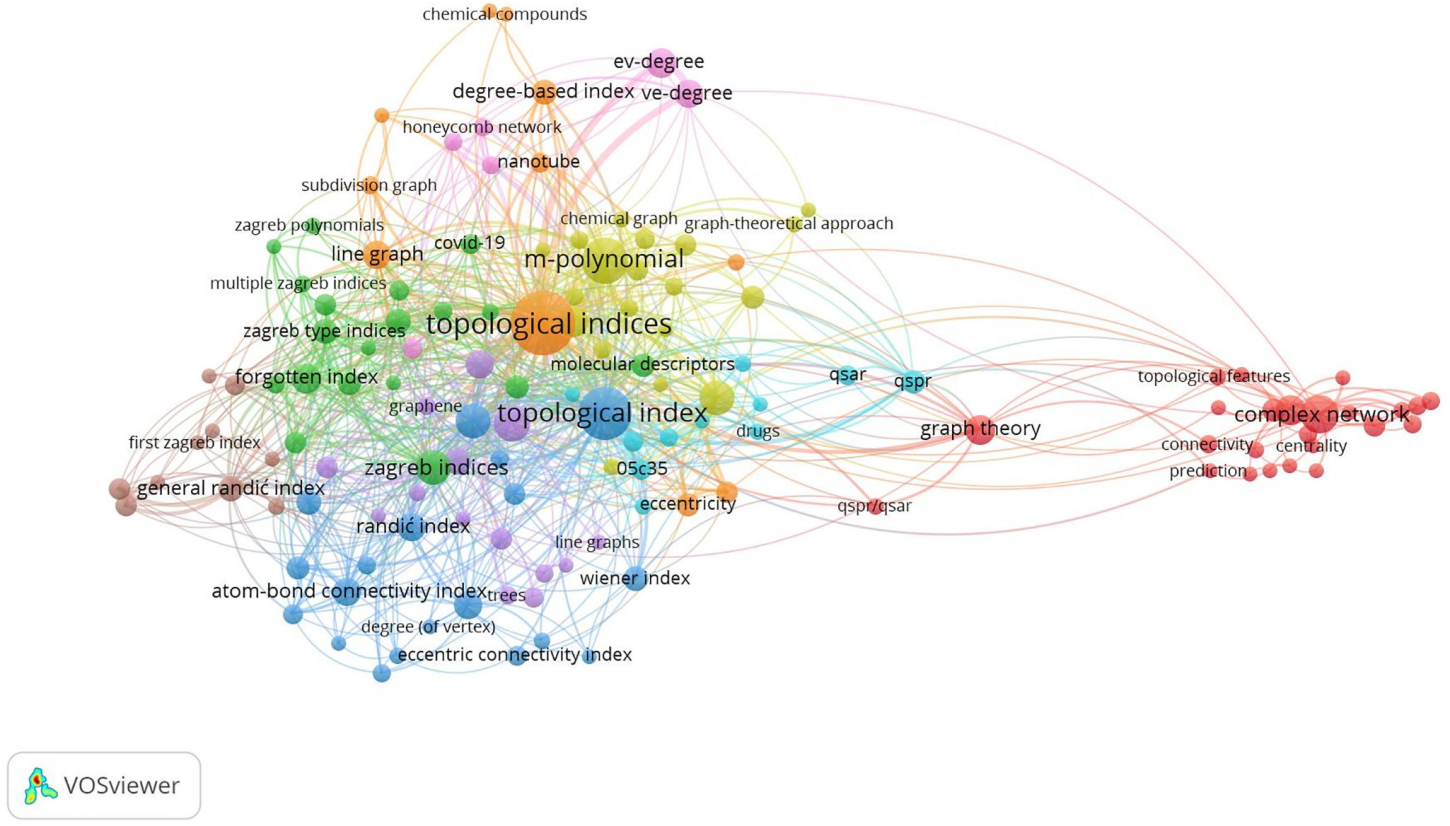
Applying bibliometrics to analyze the frequency (topological index) of a keyword across published works.

Ahmad et al. [9, 10] analyzed the theoretical study of the energy of phenylene and anthracene. Koam et al. [11] computed the valency-based topological descriptor for Hexagon Star Networks. It’s also crucial to remember that topological indices do not only describe elementary graphs. Beyond the domain of simple graphs, they find use in a variety of mathematical structures, including polynomials, matrices, and relational tables. This makes them adaptable instruments for assessing and characterizing a wide range of complex systems and networks. The variety of topological indices makes them more useful for analyzing intricate networks and systems. The bibliometric evaluation of the keywords connected to the Topological Index that we conducted is shown in Fig 1.

The Zagreb indices have attracted a lot of attention from the academic community because of their wide range of applications [12]. Of them, the Forgotten Index is a notable variant from the first class [13, 14]. This study introduced two novel types of indices, the Bi-Zagreb and Tri-Zagreb indices, using an innovative approach. These indices maintain a 1 : 1 ratio in their calculation. In this context, the degree of a vertex *y* ∈ *V*(*G*) in graph *G* is represented as Λ_*y*_. Another method that was considered was combining two different topological indices in a fractional ratio [15].

Topological indices were first developed and applied in many different fields. Wiener, H., [16] first introduced them to study the boiling temperatures of paraffin [17]. An investigation of the entropy measures of polycyclic hydroxychloroquine, for example, was carried out by Manzoor, S., et al. [18]. This work was done with a focus on the drug’s possible application in the treatment of COVID-19. Different graph entropy metrics, such as Urelement and Higher-Order Graphlets, were investigated by Huang et al. [19]. Hayat et al. [20] discussed the valency-based molecular descriptors for hydrocarbons. The entropy measurements of three different kinds of *PAHs* (Polycyclic Aromatic Hydrocarbons) were examined by Julietraja, K., et al. [21]. Mondal et al. [22] used a variety of indices to investigate the topological properties of graphene. Silicon carbide *Si*_2_*C*_3_ − *I*[*p*, *q*] double and strong double graph indices were examined by Sardar and colleagues [23]. Hayat and Imran [24] analyze the topological properties of nanocones. Entropy in relation to the Remdesivir system was the main topic of the study conducted by Feng et al. [25]. Hayat et al. [26] determined the predictive potential of distance-spectral descriptors for benzenoid hydrocarbons. Correlation and simple linear regression were covered by Zou and colleagues [27] in their study. The notion of entropy in edge-weighted graphs was first established by Chen et al. [28] in 2014. The entropy of an edge-weighted graph is described by Eq 1.

$$\begin{array}{c} E_{ϒ}\left(G\right)=-\sum_{\xi \varrho \in E(G)}\frac{\Psi (\xi^{\prime }\varrho^{\prime })}{\sum_{\xi \varrho \in E(G)}\Psi \left(\xi \varrho \right)}\;\text{log}\;[\frac{\Psi (\xi^{\prime }\varrho^{\prime })}{\sum_{\xi \varrho \in E(G)}\Psi \left(\xi \varrho \right)}] \end{array}$$ (1)

The edge weight within *G* is graphically depicted as Ψ(*ξ* *ϱ*) in this graph.

## 2 Molecular structure of Copper(II) Fluoride

**Fig 2:**
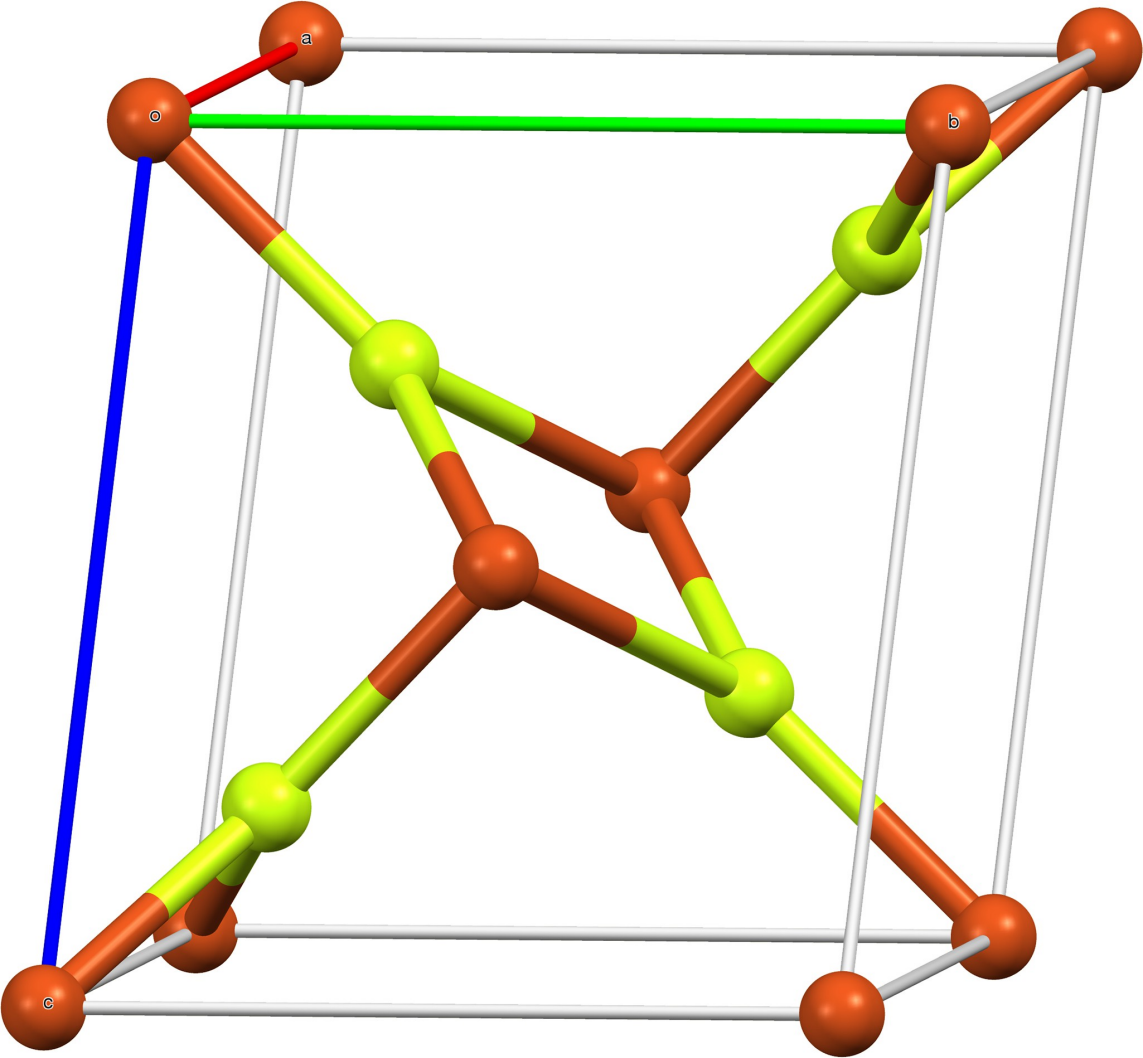
Unit cell of the anhydrous form for *CuF*_2_.

*CuF*_2_ is the chemical formula for Copper(II) Fluoride, also known as Cupric Fluoride. It consists of fluoride (F) ions and copper (Cu) ions in the +2 oxidation state. In the usual crystal lattice structure of Copper(II) Fluoride, fluoride ions surround copper ions, and vice versa. A coordination polymer, in which each Copper ion is coordinated with a certain number of fluoride ions, is a more accurate description of the crystal structure. The unique crystalline form of Copper(II) Fluoride determines the precise arrangement of atoms in the crystal lattice. It is possible for Copper(II) Fluoride to exist in many polymorphs, which means that it can adopt various crystal forms depending on the temperature and pressure. To meet the coordination preferences of Copper(II) ions, each copper ion in the crystal is surrounded by a specific number of fluoride ions. X-ray crystallography, a method for examining the arrangement of atoms within a crystal, can be used to identify the precise geometric arrangement and spacing between atoms see Fig 2.

**Fig 3:**
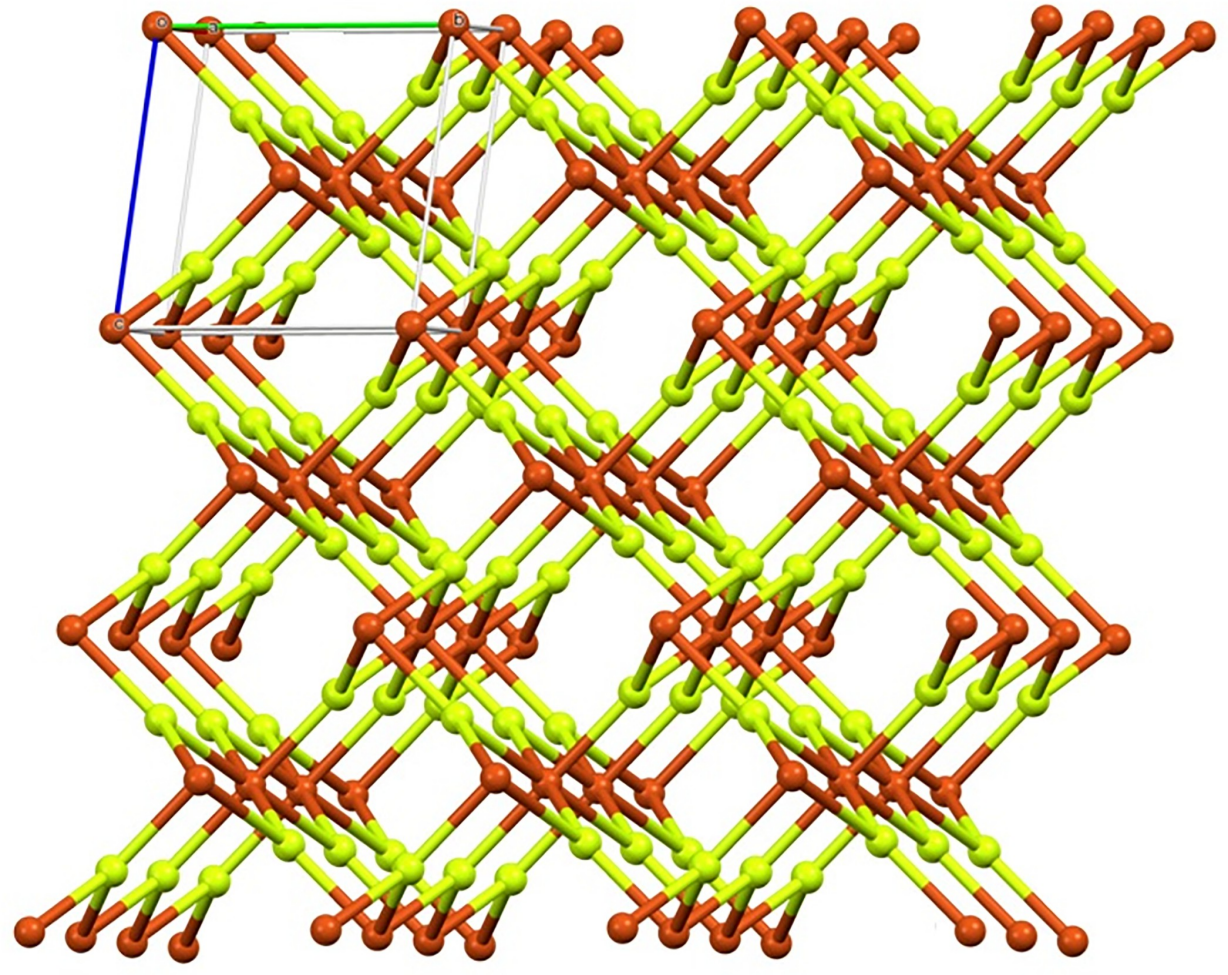
Crystal packing in the anhydrous form for *CuF*_2_.

The monoclinic crystal structure of Copper(II) Fluoride is characterized by rectangular prisms with a parallelogram base [29, 30]. The Jahn-Teller effect in d9 Copper(II) causes a distorted octahedral [4 + 2] coordination, which gives rise to the peculiar geometry. Each copper ion in this configuration is surrounded by four nearby fluoride ions at a distance of 1.93*Å*, while two further fluoride ions are located at a greater distance of 2.27*Å*. This deformation resembles the rutile structure of the d4 compound chromium(II) fluoride, or *CrF*_2_ [31, 32]. These structural distortions are brought on by the Jahn-Teller phenomenon, which also affects how the ions are arranged in space in copper(II) fluoride, giving it its unique crystal structure see Fig 3.

The crystal structure of *CuF*_2_ consists 12*mn* edges, where *m*, *n* ≥ 1 representation of the growth of the crystal structure of *CuF*_2_ horizontally and vertically respectively. More preciously, the total number of edges whose end verities have degrees (1, 3) are 2*m* + 2*n* + 2, the total number of edges whose end verities have degrees (2, 3) are 4*m* + 4*n* − 8, and the total number of edges whose end verities have degrees (3, 3) are 12*mn* − 6*m* − 6*n* + 6. Also, there are three types of edges *E*_1_, *E*_2_ and *E*_3_ in the crystal structure of *CuF*_2_ based on the degree of the vertices shown in Table 1.

**Table 1:** Edge partitioning of Copper(II) Fluoride.

Λ_*u*_, Λ_*v*_	Frequency(F)	Set of Edges
(1,3)	2*m* + 2*n* + 2	*E*_1_
(2,3)	4*m* + 4*n* − 8	*E*_2_
(3,3)	12*mn* − 6*m* − 6*n* + 6	*E*_3_

## 3 Computation of topological indices and entropy measures

Considering the previously indicated background, we carefully carried out the calculations for distinct topological indices. We carefully followed the guidelines provided for each index, accounting for the distinct features of the structures under investigation. The meticulous computation of these indices provided priceless information about how different parts interact, how their connections are arranged, and how complicated the systems under investigation are. The results of these computations greatly aided in our thorough examination, allowing us to gain a deeper understanding of the basic ideas and characteristics present in the structures we are examining.

The Bi-Zagreb index using Table 1 is computed as follows:

$$\begin{array}{ccc} BM(G) & = & \sum_{yz\in E(G)}(\left(\Lambda_{y}+\Lambda_{z}\right)+\left(\Lambda_{y}\times \Lambda_{z}\right))\\ BM(G) & = & \left(7)(2m+2n+2)+(11)(4m+4n-8)+(13)(12mn-6m-6n+6\right)\\ & = & 180mn-32m-32n+16. \end{array}$$

The Tri-Zagreb index using Table 1 is computed as follows:

$$\begin{array}{ccc} TM(G) & = & \sum_{yz\in E(G)}(\left(\Lambda_{y}^{2}+\Lambda_{z}^{2}\right)+\left(\Lambda_{y}\times \Lambda_{z}\right))\\ TM(G) & = & \left(13)(2m+2n+2)+(19)(4m+4n-8)+(27)(12mn-6m-6n+6\right)\\ & = & 324mn-60m-60n+36. \end{array}$$

The Geometric- Tri- Zagreb index using Table 1 is computed as follows:

$$\begin{array}{ccc} GTM(G) & = & \sum_{yz\in E(G)}\frac{\sqrt{\left(\Lambda_{y}\times \Lambda_{z}\right)}}{\left(\Lambda_{y}^{2}+\Lambda_{z}^{2}\right)+\left(\Lambda_{y}\times \Lambda_{z}\right)}\\ GTM(G) & = & \left(\frac{\sqrt{3}}{13}\right)\left(2m+2n+2\right)+\left(\frac{\sqrt{6}}{19}\right)\left(4m+4n-8\right)+\left(\frac{\sqrt{9}}{27}\right)\left(12mn-6m-6n+6\right)\\ & = & 1.3333mn+0.1155m+0.1155n-0.09825. \end{array}$$

The Geometric-Bi- Zagreb index using Table 1 is computed as follows:

$$\begin{array}{ccc} GBM(G) & = & \sum_{yz\in E(G)}\frac{\sqrt{\left(\Lambda_{y}\times \Lambda_{z}\right)}}{\left(\Lambda_{y}+\Lambda_{z}\right)+\left(\Lambda_{y}\times \Lambda_{z}\right)}\\ GBM(G) & = & \left(\frac{\sqrt{3}}{7}\right)\left(2m+2n+2\right)+\left(\frac{\sqrt{6}}{11}\right)\left(4m+4n-8\right)+\left(\frac{\sqrt{9}}{15}\right)\left(12mn-6m-6n+6\right)\\ & = & 2.4mn+0.1856m+0.1856n-0.0866. \end{array}$$

The Geometric-Harmonic index using Table 1 is computed as follows:

$$\begin{array}{ccc} GH(G) & = & \sum_{yz\in E(G)}\frac{\sqrt{\left(\Lambda_{y}\times \Lambda_{z}\right)}\left(\Lambda_{y}+\Lambda_{z}\right)}{2}\\ GH(G) & = & \left(\frac{\sqrt{3}\left(4\right)}{2}\right)\left(2m+2n+2\right)+\left(\frac{\sqrt{6}\left(5\right)}{2}\right)\left(4m+4n-8\right)\\ & + & \left(\frac{\sqrt{9}\left(6\right)}{2}\right)\left(12mn-6m-6n+6\right)\\ & = & 108mn-22.577m-22.577n+11.9384. \end{array}$$

The Harmonic- Tri-Zagreb index using Table 1 is computed as follows::

$$\begin{array}{ccc} HTM(G) & = & \sum_{yz\in E(G)}\frac{2}{\left(\Lambda_{y}^{2}+\Lambda_{z}^{2}\right)+\left(\Lambda_{y}\times \Lambda_{z}\right)\left(\Lambda_{y}+\Lambda_{z}\right)}\\ HTM(G) & = & \left(\frac{2}{52}\right)\left(2m+2n+2\right)+\left(\frac{2}{95}\right)\left(4m+4n-8\right)\\ & + & \left(\frac{2}{162}\right)\left(12mn-6m-6n+6\right)\\ & = & \frac{4}{27}mn+\frac{2903}{33345}m+\frac{2903}{33345}n-\frac{581}{33345}. \end{array}$$

The Harmonic- Bi- Zagreb index using Table 1 is computed as follows:

$$\begin{array}{ccc} HBM(G) & = & \sum_{yz\in E(G)}\frac{2}{\left(\Lambda_{y}+\Lambda_{z}\right)+\left(\Lambda_{y}\times \Lambda_{z}\right)\left(\left(\Lambda_{y}+\Lambda_{z}\right)\right)}\\ HBM(G) & = & \left(\frac{2}{28}\right)\left(2m+2n+2\right)+\left(\frac{2}{55}\right)\left(4m+4n-8\right)+\left(\frac{2}{90}\right)\left(12mn-6m-6n+6\right)\\ & = & \frac{4}{15}mn+\frac{179}{1155}m+\frac{179}{1155}n-\frac{17}{1155}. \end{array}$$

The Harmonic-Geometric index using Table 1 is computed as follows:

$$\begin{array}{ccc} HG(G) & = & \sum_{yz\in E(G)}\frac{2}{\sqrt{\left(\Lambda_{y}\times \Lambda_{z}\right)}\left(\left(\Lambda_{y}+\Lambda_{z}\right)\right)}\\ HG(G) & = & \left(\frac{2}{\sqrt{3}\left(4\right)}\right)\left(2m+2n+2\right)+\left(\frac{2}{\sqrt{6}\left(5\right)}\right)\left(4m+4n-8\right)\\ & + & \left(\frac{2}{\sqrt{9}\left(6\right)}\right)\left(12mn-6m-6n+6\right)\\ & = & 1.3333mn+0.56391m+0.56391n-0.06235. \end{array}$$

The Bi- Zagreb- Harmonic index using Table 1 is computed as follows:

$$\begin{array}{ccc} BMH(G) & = & \sum_{yz\in E(G)}\frac{\left(\Lambda_{y}+\Lambda_{z}\right)+\left(\Lambda_{y}\times \Lambda_{z}\right)\left(\Lambda_{y}+\Lambda_{z}\right)}{2}\\ BMH(G) & = & \left(\frac{28}{2}\right)\left(2m+2n+2\right)+\left(\frac{55}{2}\right)\left(4m+4n-8\right)+\left(\frac{90}{2}\right)\left(12mn-6m-6n+6\right)\\ & = & 540mn-132m-132n+78. \end{array}$$

The Bi- Zagreb Geometric index using Table 1 is computed as follows:

$$\begin{array}{ccc} BMG(G) & = & \sum_{yz\in E(G)}\frac{\left(\Lambda_{y}+\Lambda_{z}\right)+\left(\Lambda_{y}\times \Lambda_{z}\right)}{\sqrt{\left(\Lambda_{y}\times \Lambda_{z}\right)}}\\ BMG(G) & = & \left(\frac{7}{\sqrt{3}}\right)\left(2m+2n+2\right)+\left(\frac{11}{\sqrt{6}}\right)\left(4m+4n-8\right)+\left(\frac{15}{\sqrt{9}}\right)\left(12mn-6m-6n+6\right)\\ & = & 60mn-3.954m-3.954n+2.158. \end{array}$$

The Tri- Zagreb Harmonic index using Table 1 is computed as follows:

$$\begin{array}{ccc} TMH(G) & = & \sum_{yz\in E(G)}\frac{\left(\Lambda_{y}^{2}+\Lambda_{z}^{2}\right)+\left(\Lambda_{y}\times \Lambda_{z}\right)\left(\Lambda_{y}+\Lambda_{z}\right)}{2}\\ TMH(G) & = & \left(\frac{52}{2}\right)\left(2m+2n+2\right)+\left(\frac{95}{2}\right)\left(4m+4n-8\right)+\left(\frac{162}{2}\right)\left(12mn-6m-6n+6\right)\\ & = & 972mn-244m-244n+158. \end{array}$$

The Tri- Zagreb Geometric index using Table 1 is computed as follows:

$$\begin{array}{ccc} TMG(G) & = & \sum_{yz\in E(G)}\frac{\left(\Lambda_{y}^{2}+\Lambda_{z}^{2}\right)+\left(\Lambda_{y}\times \Lambda_{z}\right)}{\sqrt{\left(\Lambda_{y}\times \Lambda_{z}\right)}}\\ TMG(G) & = & \left(\frac{13}{\sqrt{3}}\right)\left(2m+2n+2\right)+\left(\frac{19}{\sqrt{6}}\right)\left(4m+4n-8\right)+\left(\frac{27}{\sqrt{9}}\right)\left(12mn-6m-6n+6\right)\\ & = & 108mn-7.962m-7.962n+6.956. \end{array}$$

**Fig 4:**
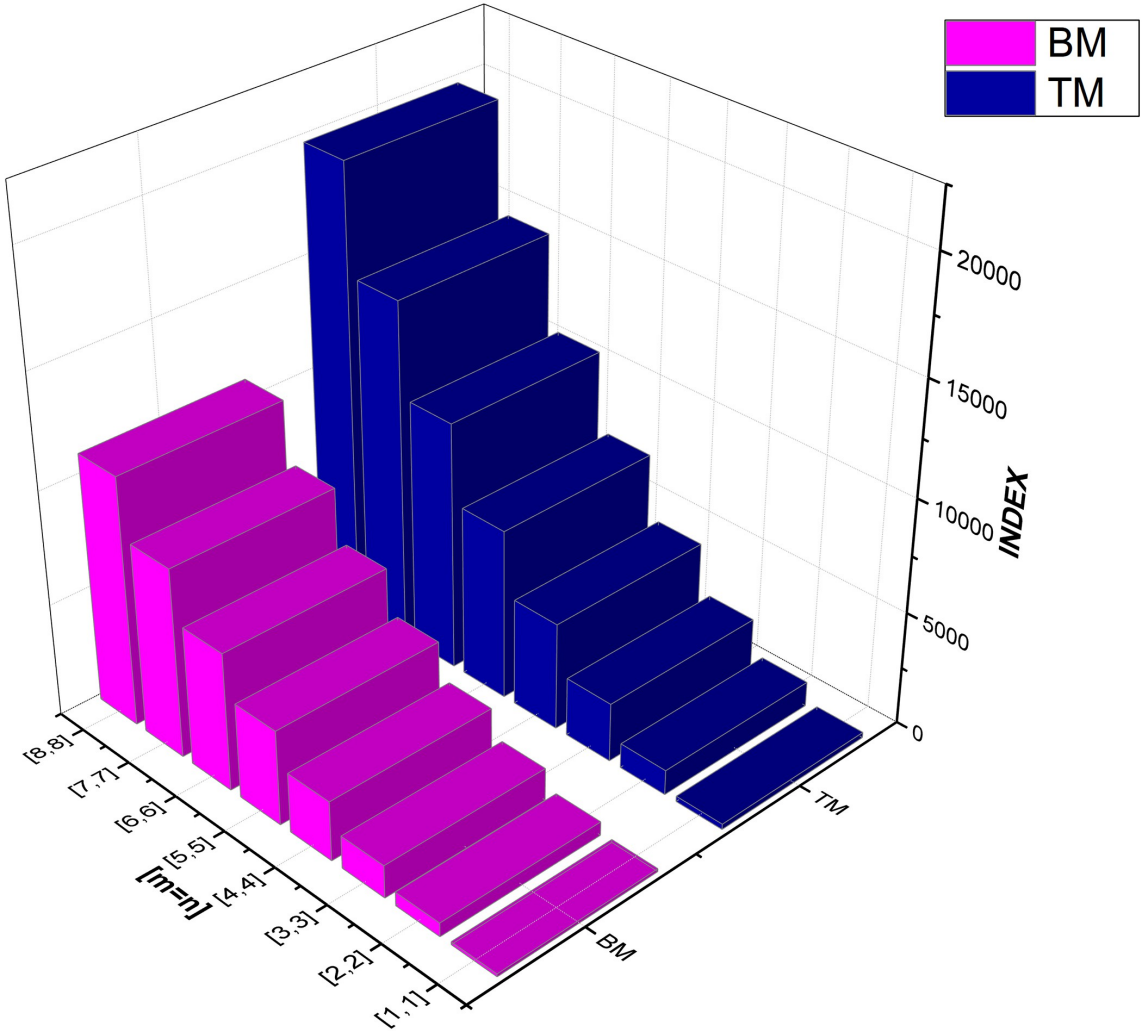
Graphical comparison between BM, TM.

**Fig 5:**
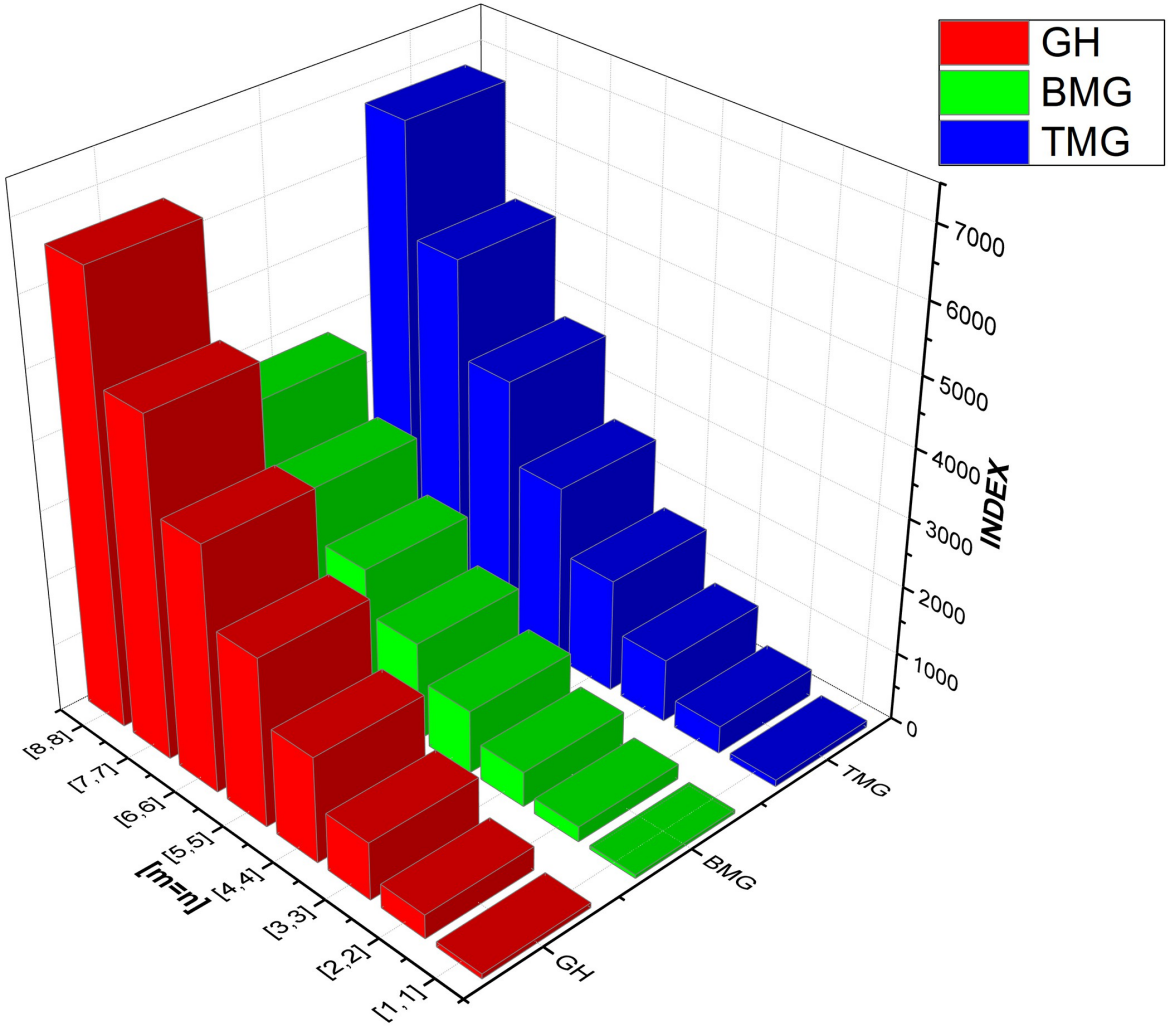
Graphical comparison between GH, BMG, TMG.

**Fig 6:**
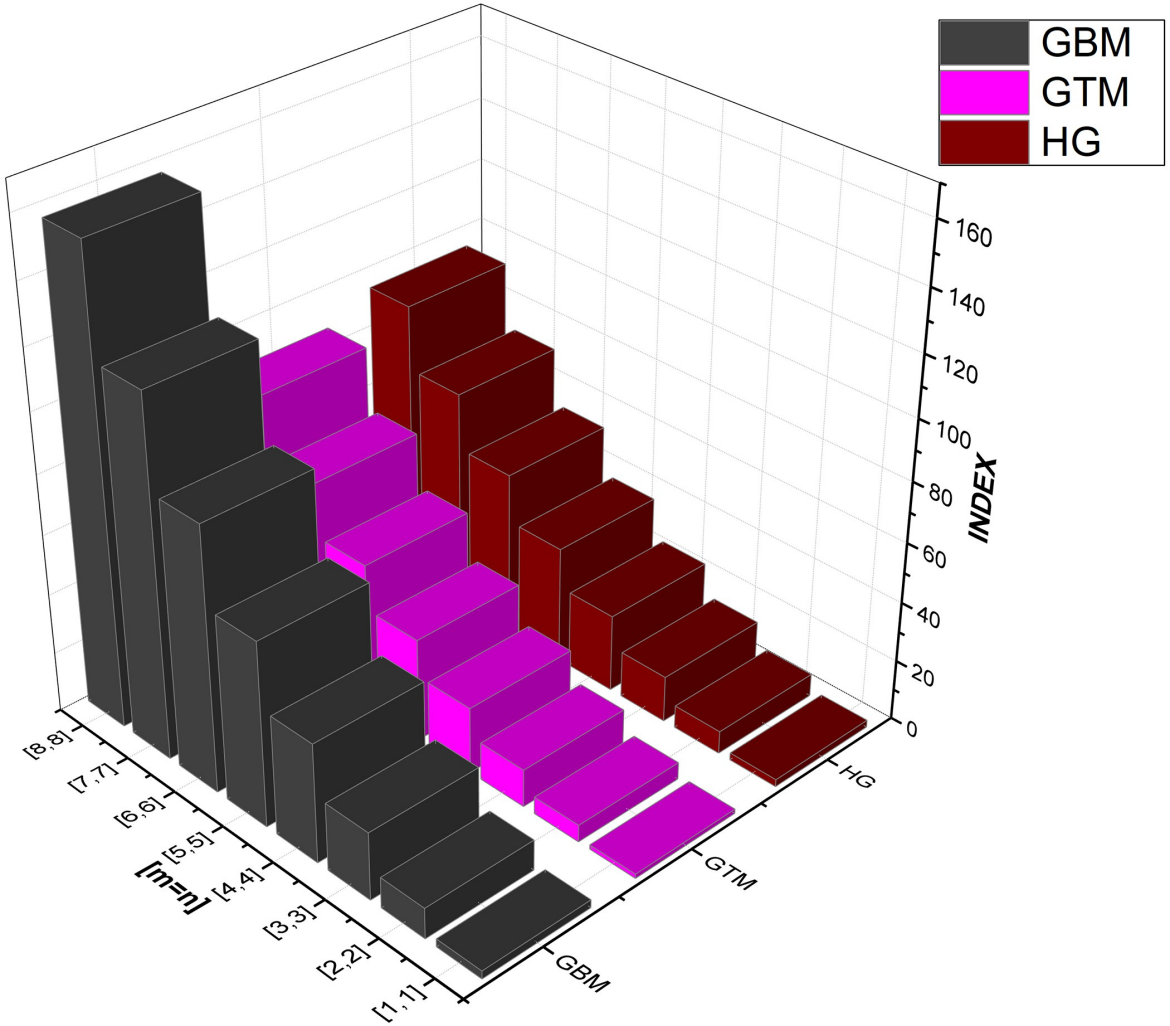
Graphical comparison between GBM, GTM, HG.

**Fig 7:**
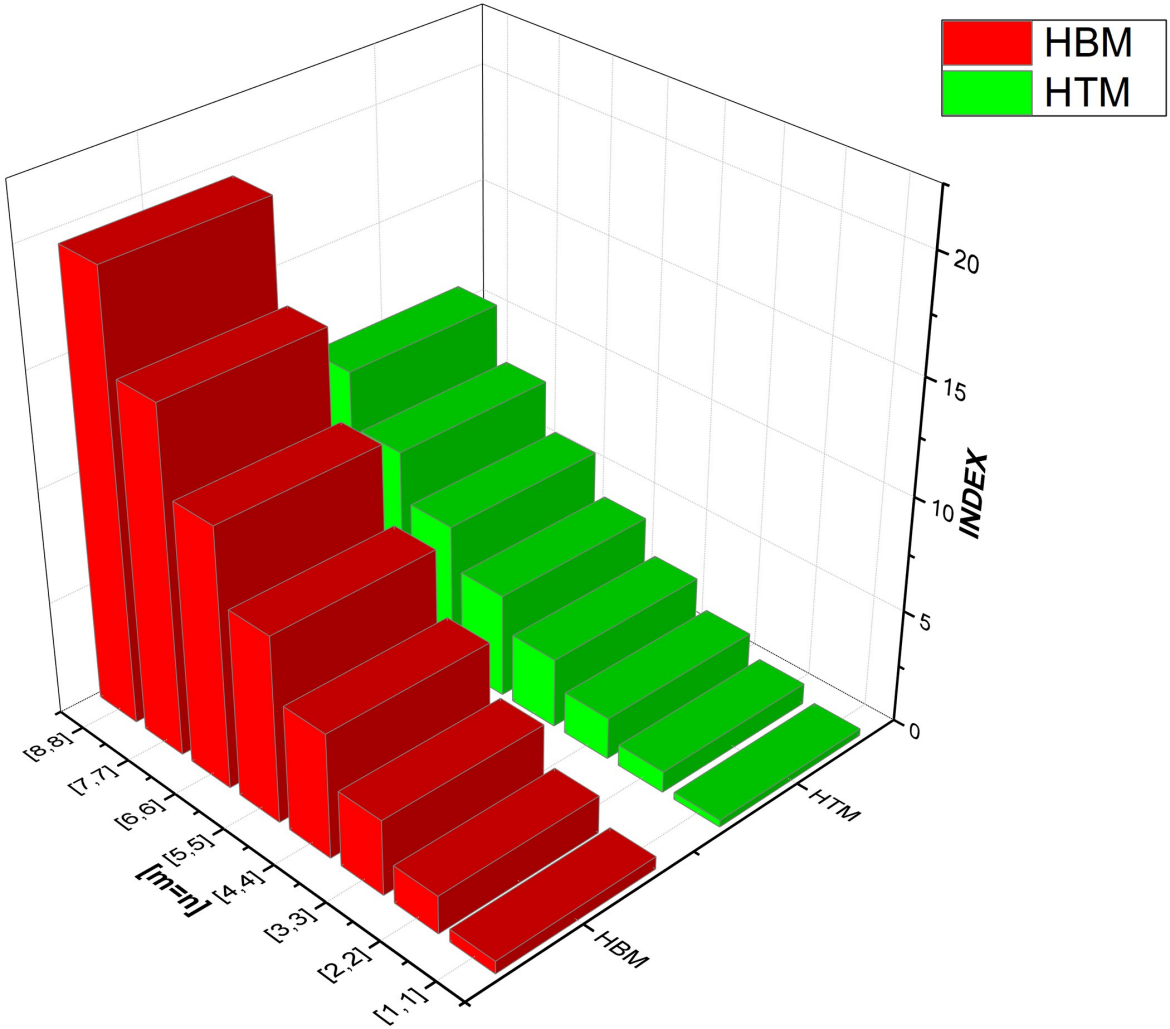
Graphical comparison between HBM, HTM.

**Fig 8:**
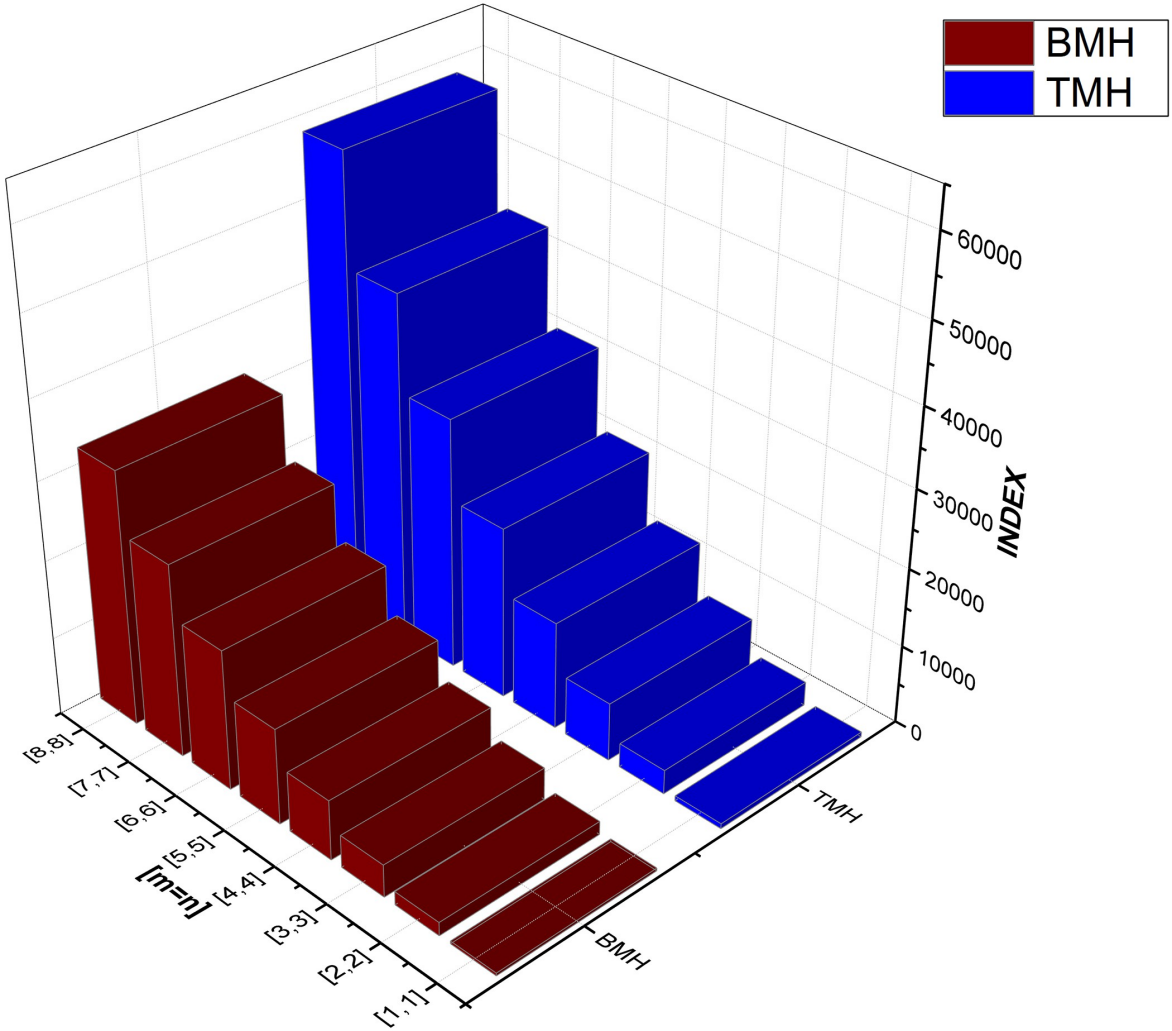
Graphical comparison between BMH and TMH.

As numerical descriptors in the field of graph theory, topological indices are mostly used to explain the structural features of molecules. They are essential to understanding the behavior and operation of biological and chemical systems. Researchers are better equipped to understand the system they are studying because of the accurate calculation of these numerical indexes. Our emphasis on degree-based indices customized for *CuF*_2_ makes this method very beneficial. To provide a thorough examination of system features, we have also included Table 2, which examines the performance of these degree-based indices at various values of *m* and *n*. The data in Table 2 clearly shows that the indices show an increase in tandem with the increases in *m* and *n* values. For more details see Figs 4–8 for a graphic depiction of this pattern.

**Table 2:** Numerical comparative evaluation of distinct computed indices.

Index	[1, 1]	[2, 2]	[3, 3]	[4, 4]	[5, 5]	[6, 6]
*BM*(*G*)	132	608	1444	2640	4196	6112
*TM*(*G*)	240	1092	2592	4740	7536	10980
*GH*(*G*)	74.7844	353.6304	848.4764	1559.3224	2486.1684	3629.0144
*GBM*(*G*)	2.6846	10.2558	22.627	39.7982	61.7694	88.5406
*GTM*(*G*)	1.46605	5.6970	12.5945	22.1586	34.3893	49.2866
*HG*(*G*)	2.3987	7.5264	15.3207	25.7816	38.9091	54.7032
*HBM*(*G*)	0.5619	1.6719	3.3152	5.4918	8.2017	111.4450
*HTM*(*G*)	0.3048	0.9234	1.8383	3.0494	4.5569	6.3606
*BMG*(*G*)	54.25	226.342	518.434	930.526	1462.618	2114.71
*BMH*(*G*)	354	1710	4146	7662	12258	17934
*TMG*(*G*)	99.032	407.108	931.184	1671.26	2627.336	3799.412
*TMH*(*G*)	642	3070	7442	13758	22018	32222

As the topological indices go from [1, 1] to [6, 6], their values show a clear and consistent increasing trend. As illustrated by the growing index values, this tendency reflects a proportional rise in disorder or unpredictability within the system as it grows in complexity or scale. This observation supports the concept that larger systems often contain a greater number of microstates, resulting in higher topological index values.

Entropy measures are widely used in data analysis, thermodynamics, and information theory, and they are useful tools for quantifying the degree of uncertainty or information contained in a dataset. They are critical in unraveling the complexities and dispersion of data, providing researchers with critical insights into the system under investigation. We computed a variety of standard entropy metrics in a methodical manner. This analytical method helped us to gain a more complete knowledge of the underlying data and identify the patterns driving information flow inside the graph. The extensive use of entropy analysis was critical in revealing the intricate structure and complexities of the edge-weighted graph.

When *G* ≅ *CuF*_2_, the entropy of the Bi-Zagreb index can be determined by using Table 1 and Eq 2 into Eq 1.

$$\begin{array}{ccc} ENTBM(G) & = & \text{log}\left(180mn-32m-32n+16\right)-\frac{\left(2m+2n+2)(7)\;\text{log}(7\right)}{\left(180mn-32m-32n+16\right)}\\ & - & \frac{\left(4m+4n-8)(11)\;\text{log}(11\right)}{\left(180mn-32m-32n+16\right)}-\frac{\left(12mn-6m-6n+6)(15)\;\text{log}(15\right)}{\left(180mn-32m-32n+16\right)} \end{array}$$

When *G* ≅ *CuF*_2_, the entropy of the Tri-Zagreb index can be determined by using Table 1 and Eq 2 into Eq 1.

$$\begin{array}{ccc} ENTTM(G) & = & \text{log}\left(324mn-60m-60n+36\right)-\frac{\left(2m+2n+2)(13)\;\text{log}(13\right)}{\left(324mn-60m-60n+36\right)}\\ & - & \frac{\left(4m+4n-8)(19)\;\text{log}(19\right)}{\left(324mn-60m-60n+36\right)}-\frac{\left(12mn-6m-6n+6)(27)\;\text{log}(27\right)}{\left(324mn-60m-60n+36\right)} \end{array}$$

When *G* ≅ *CuF*_2_, the entropy of the Geometric Tri-Zagreb index can be determined by using Table 1 and Eq 2 into Eq 1.

$$\begin{array}{ccc} ENTGTM(G) & = & \text{log}(1.3333mn+0.1155m+0.1155n-0.09825)\\ & - & \frac{\left(2m+2n+2\right)\left(\frac{\sqrt{3}}{13}\right)\;\text{log}\left(\frac{\sqrt{3}}{13}\right)}{\left(1.3333mn+0.1155m+0.1155n-0.09825\right)}\\ & - & \frac{\left(4m+4n-8\right)\left(\frac{\sqrt{6}}{19}\right)\;\text{log}\left(\frac{\sqrt{6}}{19}\right)}{\left(1.3333mn+0.1155m+0.1155n-0.09825\right)}\\ & - & \frac{\left(12mn-6m-6n+6\right)\left(\frac{\sqrt{9}}{27}\right)\;\text{log}\left(\frac{\sqrt{9}}{27}\right)}{\left(1.3333mn+0.1155m+0.1155n-0.09825\right)} \end{array}$$

When *G* ≅ *CuF*_2_, the entropy of the Geometric Bi-Zagreb index can be determined by using Table 1 and Eq 2 into Eq 1.

$$\begin{array}{ccc} ENTGBM(G) & = & \text{log}(2.4mn+0.1856m+0.1856n-0.0866)\\ & - & \frac{\left(2m+2n+2\right)\left(\frac{\sqrt{3}}{7}\right)\;\text{log}\left(\frac{\sqrt{3}}{7}\right)}{\left(2.4mn+0.1856m+0.1856n-0.0866\right)}\\ & - & \frac{\left(4m+4n-8\right)\left(\frac{\sqrt{6}}{11}\right)\;\text{log}\left(\frac{\sqrt{6}}{11}\right)}{\left(2.4mn+0.1856m+0.1856n-0.0866\right)}\\ & - & \frac{\left(12mn-6m-6n+6\right)\left(\frac{\sqrt{9}}{15}\right)\;\text{log}\left(\frac{\sqrt{9}}{15}\right)}{\left(2.4mn+0.1856m+0.1856n-0.0866\right)} \end{array}$$

When *G* ≅ *CuF*_2_, the entropy of the Geometric Harmonic index can be determined by using Table 1 and Eq 2 into Eq 1.

$$\begin{array}{ccc} ENTGH(G) & = & \text{log}(108mn-22.577m-22.577n+11.938)\\ & - & \frac{\left(2m+2n+2\right)\left(\frac{\sqrt{3}\left(4\right)}{2}\right)\;\text{log}\left(\frac{\sqrt{3}\left(4\right)}{2}\right)}{\left(108mn-22.577m-22.577n+11.938\right)}\\ & - & \frac{\left(4m+4n-8\right)\left(\frac{\sqrt{6}\left(5\right)}{2}\right)\;\text{log}\left(\frac{\sqrt{6}\left(5\right)}{2}\right)}{\left(108mn-22.577m-22.577n+11.938\right)}\\ & - & \frac{\left(12mn-6m-6n+6\right)\left(\frac{\sqrt{9}\left(6\right)}{2}\right)\;\text{log}\left(\frac{\sqrt{9}\left(6\right)}{2}\right)}{\left(108mn-22.577m-22.577n+11.938\right)} \end{array}$$

When *G* ≅ *CuF*_2_, the entropy of the Harmonic Tri- Zagreb index can be determined by using Table 1 and Eq 2 into Eq 1.

$$\begin{array}{ccc} ENTHTM(G) & = & \text{log}(\frac{4}{27}mn+\frac{2903}{33345}m+\frac{2903}{33345}n-\frac{581}{33345})\\ & - & \frac{\left(2m+2n+2\right)\left(\frac{2}{52}\right)\;\text{log}\left(\frac{2}{52}\right)}{\left(\frac{4}{27}mn+\frac{2903}{33345}m+\frac{2903}{33345}n-\frac{581}{33345}\right)}\\ & - & \frac{\left(4m+4n-8\right)\left(\frac{2}{95}\right)\;\text{log}\left(\frac{2}{95}\right)}{\left(\frac{4}{27}mn+\frac{2903}{33345}m+\frac{2903}{33345}n-\frac{581}{33345}\right)}\\ & - & \frac{\left(12mn-6m-6n+6\right)\left(\frac{2}{162}\right)\;\text{log}\left(\frac{2}{162}\right)}{\left(\frac{4}{27}mn+\frac{2903}{33345}m+\frac{2903}{333445}n-\frac{581}{33345}\right)} \end{array}$$

When *G* ≅ *CuF*_2_, the entropy of the Harmonic Bi-Zagreb index can be determined by using Table 1 and Eq 2 into Eq 1.

$$\begin{array}{ccc} ENTHBM(G) & = & \text{log}(0.26667mn+0.15498m+0.15498n-0.014719)\\ & - & \frac{\left(2m+2n+2\right)\left(\frac{2}{28}\right)\;\text{log}\left(\frac{2}{28}\right)}{\left(0.26667mn+0.15498m+0.15498n-0.014719\right)}\\ & - & \frac{\left(4m+4n-8\right)\left(\frac{2}{55}\right)\;\text{log}\left(\frac{2}{55}\right)}{\left(0.26667mn+0.15498m+0.15498n-0.014719\right)}\\ & - & \frac{\left(12mn-6m-6n+6\right)\left(\frac{2}{90}\right)\;\text{log}\left(\frac{2}{90}\right)}{\left(0.26667mn+0.15498m+0.15498n-0.014719\right)} \end{array}$$

When *G* ≅ *CuF*_2_, the entropy of the Harmonic Geometric index can be determined by using Table 1 and Eq 2 into Eq 1.

$$\begin{array}{ccc} ENTHG(G) & = & \text{log}(1.3333mn+0.5639m+0.5639n-0.06235)\\ & - & \frac{\left(2m+2n+2\right)\left(\frac{2}{\sqrt{3}\left(4\right)}\right)\;\text{log}\left(\frac{2}{\sqrt{3}\left(4\right)}\right)}{\left(1.3333mn+0.5639m+0.5639n-0.06235\right)}\\ & - & \frac{\left(4m+4n-8\right)\left(\frac{2}{\sqrt{6}\left(5\right)}\right)\;\text{log}\left(\frac{2}{\sqrt{6}\left(5\right)}\right)}{\left(1.3333mn+0.5639m+0.5639n-0.06235\right)}\\ & - & \frac{\left(12mn-6m-6n+6\right)\left(\frac{2}{\sqrt{9}\left(6\right)}\right)\;\text{log}\left(\frac{2}{\sqrt{9}\left(6\right)}\right)}{\left(1.3333mn+0.5639m+0.5639n-0.06235\right)} \end{array}$$

When *G* ≅ *CuF*_2_, the entropy of the Bi-Zagreb Harmonic index can be determined by using Table 1 and Eq 2 into Eq 1.

$$\begin{array}{ccc} ENTBMH(G) & = & \text{log}\left(540mn-132m-132n+78\right)-\frac{\left(2m+2n+2\right)\left(\frac{28}{2}\right)\;\text{log}\left(\frac{28}{2}\right)}{\left(540mn-132m-132n+78\right)}\\ & - & \frac{\left(4m+4n-8\right)\left(\frac{55}{2}\right)\;\text{log}\left(\frac{55}{2}\right)}{\left(540mn-132m-132n+78\right)}-\frac{\left(12mn-6m-6n+6\right)\left(\frac{90}{2}\right)\;\text{log}\left(\frac{90}{2}\right)}{\left(540mn-132m-132n+78\right)} \end{array}$$

When *G* ≅ *CuF*_2_, the entropy of the Bi-Zagreb Geometric index can be determined by using Table 1 and Eq 2 into Eq 1.

$$\begin{array}{ccc} ENTBMG(G) & = & \text{log}(60mn-3.954m-3.954n+2.158)\\ & - & \frac{\left(2m+2n+2\right)\left(\frac{7}{\sqrt{3}}\right)\;\text{log}\left(\frac{7}{\sqrt{3}}\right)}{\left(60mn-3.954m-3.954n+2.158\right)}\\ & - & \frac{\left(4m+4n-8\right)\left(\frac{11}{\sqrt{6}}\right)\;\text{log}\left(\frac{11}{\sqrt{6}}\right)}{\left(60mn-3.954m-3.954n+2.158\right)}\\ & - & \frac{\left(12mn-6m-6n+6\right)\left(\frac{15}{\sqrt{9}}\right)\;\text{log}\left(\frac{15}{\sqrt{9}}\right)}{\left(60mn-3.954m-3.954n+2.158\right)} \end{array}$$

When *G* ≅ *CuF*_2_, the entropy of the Tri-Zagreb Harmonic index can be determined by using Table 1 and Eq 2 into Eq 1.

$$\begin{array}{ccc} ENTTMH(G) & = & \text{log}(972mn-244m-244n+158)\\ & - & \frac{\left(2m+2n+2\right)\left(\frac{52}{2}\right)\;\text{log}\left(\frac{52}{2}\right)}{\left(972mn-244m-244n+158\right)}\\ & - & \frac{\left(4m+4n-8\right)\left(\frac{95}{2}\right)\;\text{log}\left(\frac{95}{2}\right)}{\left(972mn-244m-244n+158\right)}\\ & - & \frac{\left(12mn-6m-6n+6\right)\left(\frac{162}{2}\right)\;\text{log}\left(\frac{162}{2}\right)}{\left(972mn-244m-244n+158\right)} \end{array}$$

When *G* ≅ *CuF*_2_, the entropy of the Tri-Zagreb Geometric index can be determined by using Table 1 and Eq 2 into Eq 1.

$$\begin{array}{ccc} ENTTMG(G) & = & \text{log}(108mn-7.962m-7.962n+6.956)\\ & - & \frac{\left(2m+2n+2\right)\left(\frac{13}{\sqrt{3}}\right)\;\text{log}\left(\frac{13}{\sqrt{3}}\right)}{\left(108mn-7.962m-7.962n+6.956\right)}\\ & - & \frac{\left(4m+4n-8\right)\left(\frac{19}{\sqrt{6}}\right)\;\text{log}\left(\frac{19}{\sqrt{6}}\right)}{\left(108mn-7.962m-7.962n+6.956\right)}\\ & - & \frac{\left(12mn-6m-6n+6\right)\left(\frac{27}{\sqrt{9}}\right)\;\text{log}\left(\frac{27}{\sqrt{9}}\right)}{\left(108mn-7.962m-7.962n+6.956\right)} \end{array}$$

**Fig 9:**
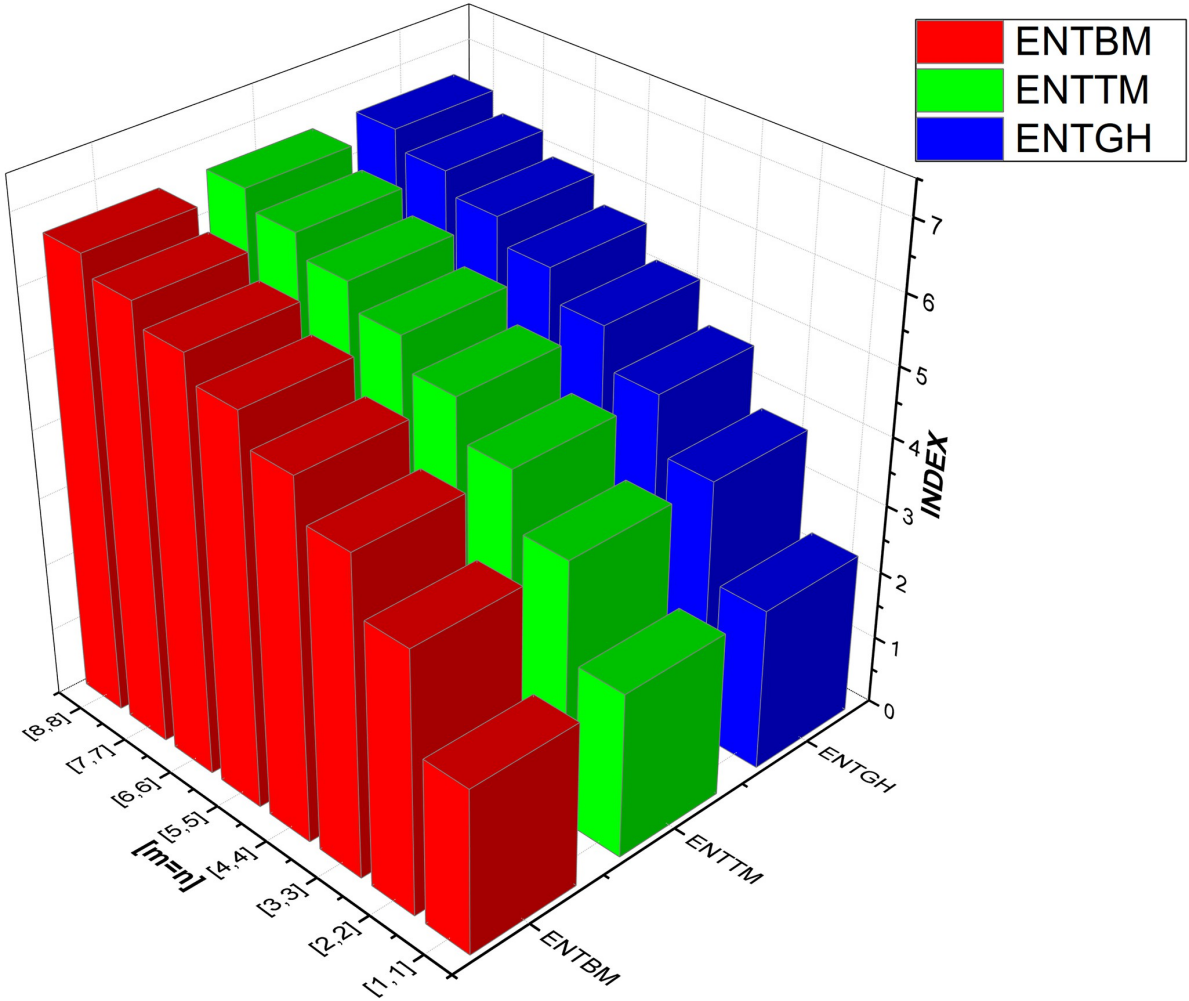
Graphical comparison between ENTBM, ENTTM, ENTGH.

**Fig 10:**
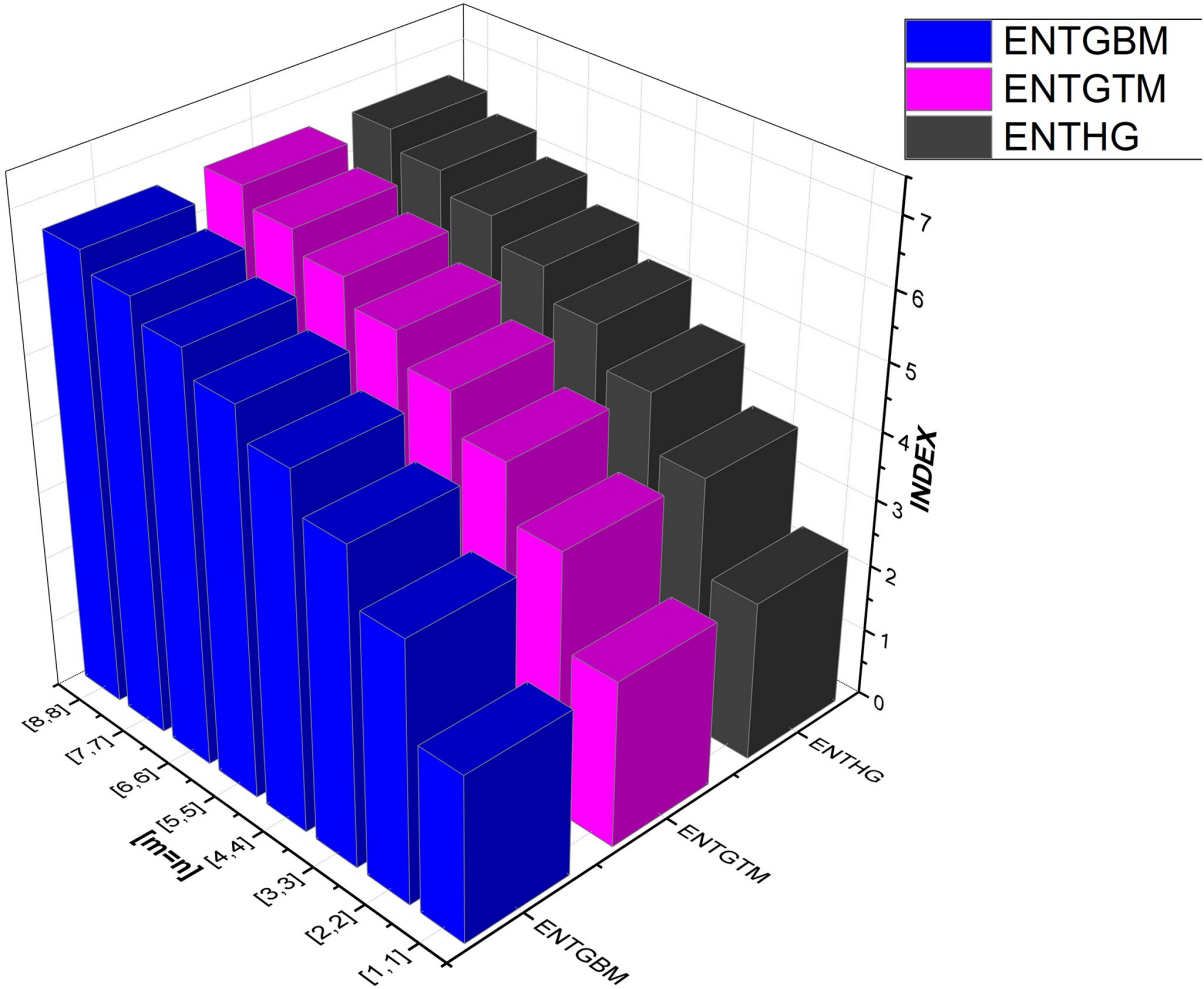
Graphical comparison between ENTGBM, ENTGTM, ENTHG.

**Fig 11:**
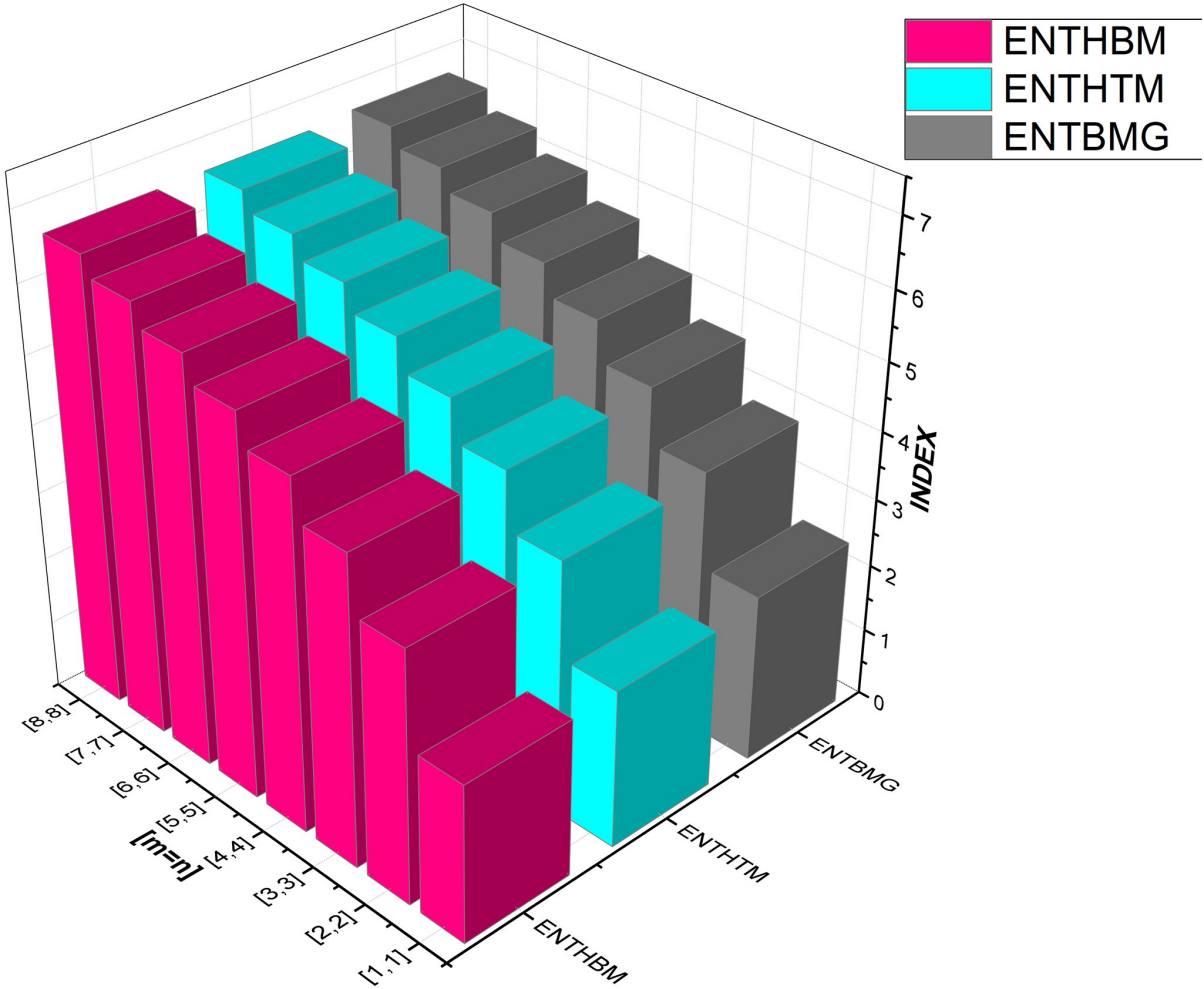
Graphical comparison between ENTHBM, ENTHTM, ENTBMG.

**Fig 12:**
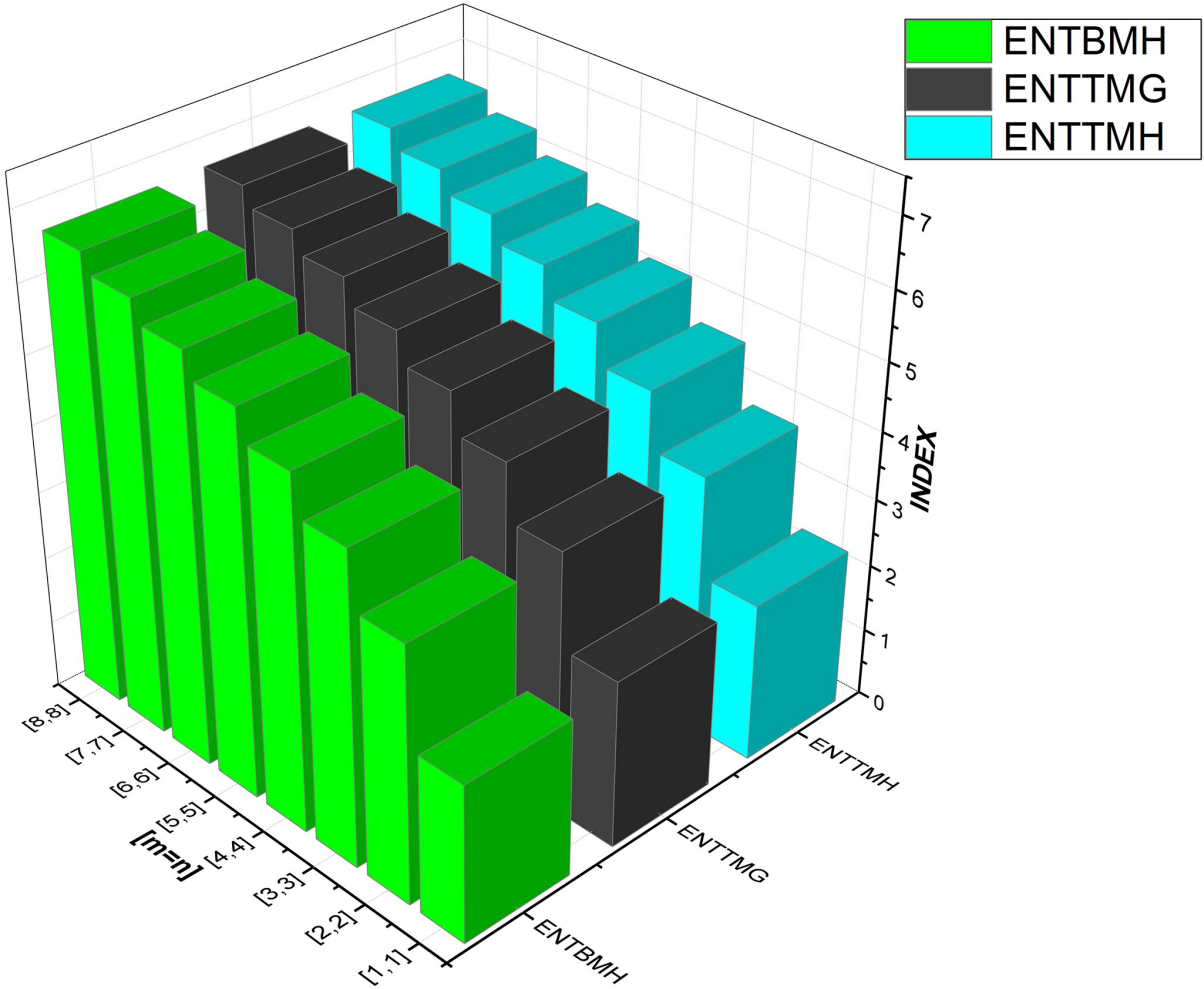
Graphical comparison between ENTBMH, ENTTMG, ENTTMH.

We have also included Table 3 to examine the differences in edge weight entropy across different *m* and *n* values in order to undertake a thorough examination of the system’s characteristics. Figs 9–12 provide graphical representations of these dynamics for ease of understanding.

**Table 3:** Comparative entropy analysis for various indices.

Index	[1, 1]	[2, 2]	[3, 3]	[4, 4]	[5, 5]	[6, 6]
*ENT*_*BM*_	2.4172	3.8348	4.6566	5.2378	5.6878	6.0550
*ENT*_*TM*_	2.4223	3.8359	4.6570	5.2379	5.6878	6.0548
*ENT*_*GH*_	2.3828	3.8180	4.6452	5.2292	5.6809	6.0492
*ENT*_*GBM*_	2.4792	3.8672	4.6790	5.2550	5.7017	6.0667
*ENT*_*GTM*_	2.4808	3.8678	4.6793	5.2550	5.7017	6.0667
*ENT*_*HG*_	2.3828	3.7825	4.6089	5.1960	5.6509	6.0222
*ENT*_*HBM*_	2.3396	3.7362	4.5666	5.1585	5.6177	5.9924
*ENT*_*HTM*_	2.3452	3.7431	4.5715	5.1617	5.6197	5.9937
*ENT*_*BMG*_	2.4793	3.8676	4.6794	5.2553	5.7020	6.0669
*ENT*_*BMH*_	2.3397	3.7964	4.6306	5.2181	5.6719	6.0416
*ENT*_*TMG*_	2.4807	3.8680	4.6796	5.2553	5.7020	6.0669
*ENT*_*TMH*_	2.3463	3.7973	4.6305	5.2179	5.6717	6.0409

## 4 Topology of the networks of indices and entropies

In this section, we have inferred two networks based on a similarity measure given in (2). This similarity measure was introduced by Inber et al. in [33]. The similarity measure in (2) is based on Pearson correlation and Euclidean distance. The main objective behind the construction of such a measure was to capture the highly similar variables in a data set and infer a network among them. This measure isolates the groups of features from each other which have weak connections among them, while the variables having high similarity are grouped together making a cluster. The obtained network is a disjoint graph consisting of clusters of highly similar variables.

$$\begin{array}{c} S=sign\left(\varpi \left(X\right)\right)\times \frac{\left|\varpi \left(X\right)\right|+\left(1-\frac{log(\nu (X)+1)}{max(log(\nu (X)+1))}\right)}{2}, \end{array}$$ (2)

In (2), *X* denotes the matrix of data, *ϖ* and *ν* are the correlation and distance functions defined on the data matrix, respectively. The values lie from −1 to 1 where a value near 1 predicts highly positively related objects while −1 shows high dissimilarity between the objects. *sign* function is used to capture the sign of the relation between objects. This measure is implemented in this paper to capture stronger variables in the set of indices and in the set of entropies. To make the similarity measure stricter the obtained matrix is converted into an adjacency matrix by taking a power transformation. This adjacency matrix is further utilized in the ‘*igraph*’ package in R to construct a network. We have two data sets namely ‘Indices’ and ‘Entropy’ comprising of all the indices and all the entropies, respectively which are computed above. Each data set is comprised of 12 variables and 8 observations. This work is done in R using the libraries ‘*readr*’, ‘*RColorBrewer*’, ‘*gplots*’, ‘*ggplot2*’, ‘*knitr*’, ‘*reshape2*’, ‘*WGCNA*’. *WGCNA* is a package that is specially designed to capture such kinds of networks and detect modules in the field of systems biology, for details see [34].

**Fig 13:**
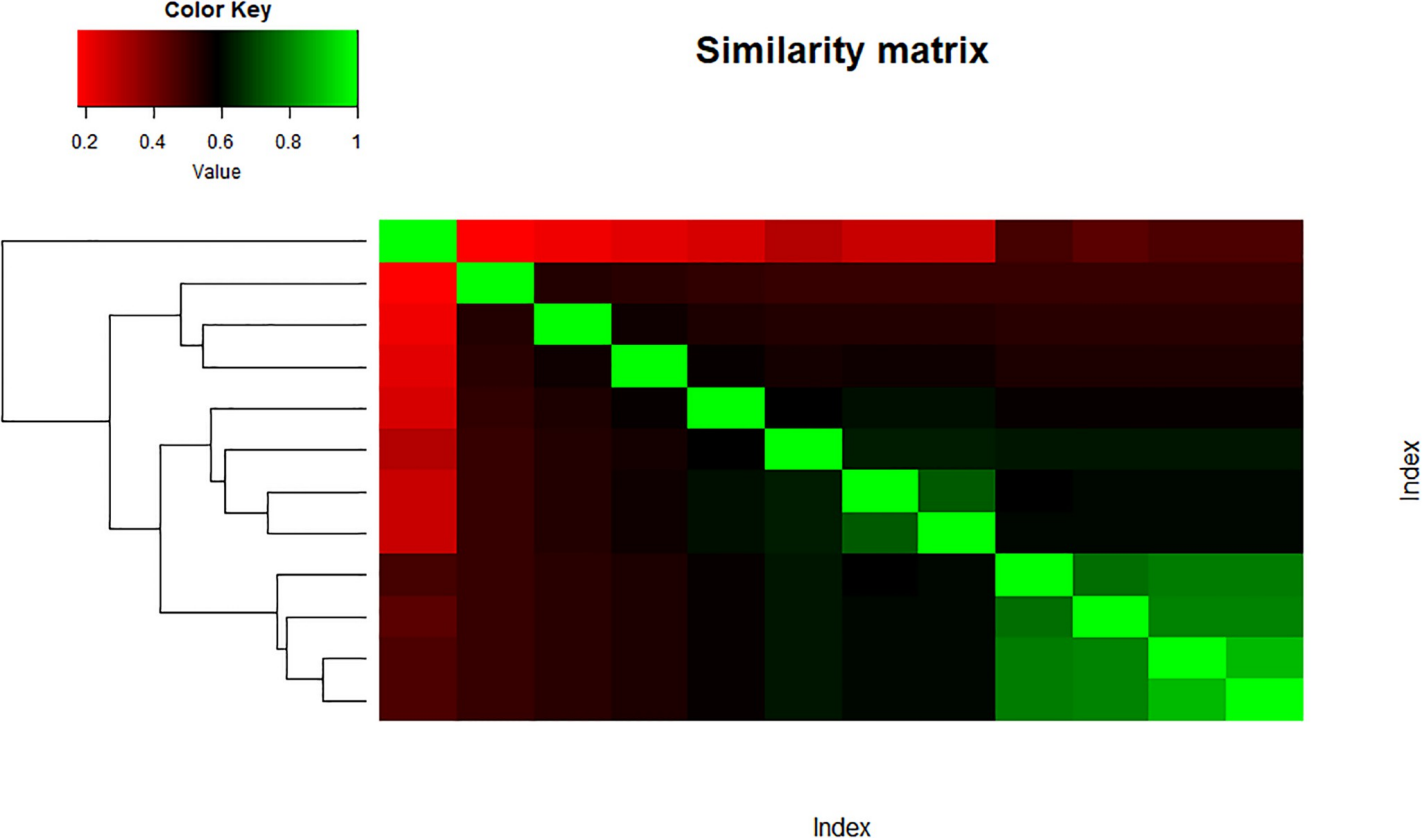
Similarity index matrix.

**Fig 14:**
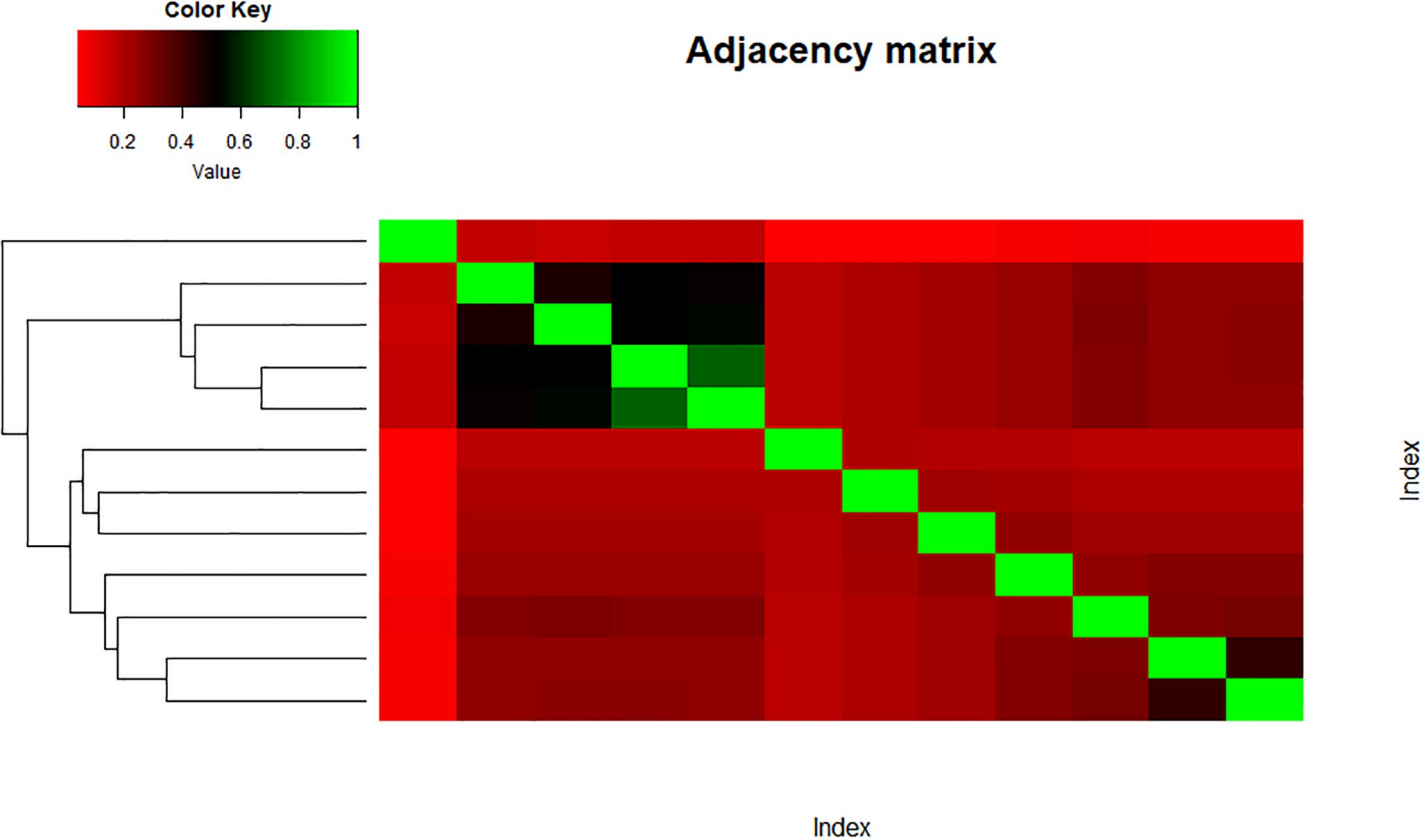
Adjacency index matrix.

The main objective of the paper is to see whether the structure or the topology of the network is preserved after taking entropies of the indices or not. It might show a good sign of connectedness between variables of both data sets which could suggest good mathematical connections among them. Figs 13 and 14 represent the heatmap of the similarity matrix and adjacency matrix of the set ‘Indices’, respectively. The green cells in the heatmap show highly similar variables while the red part shows low similarity.

**Fig 15:**
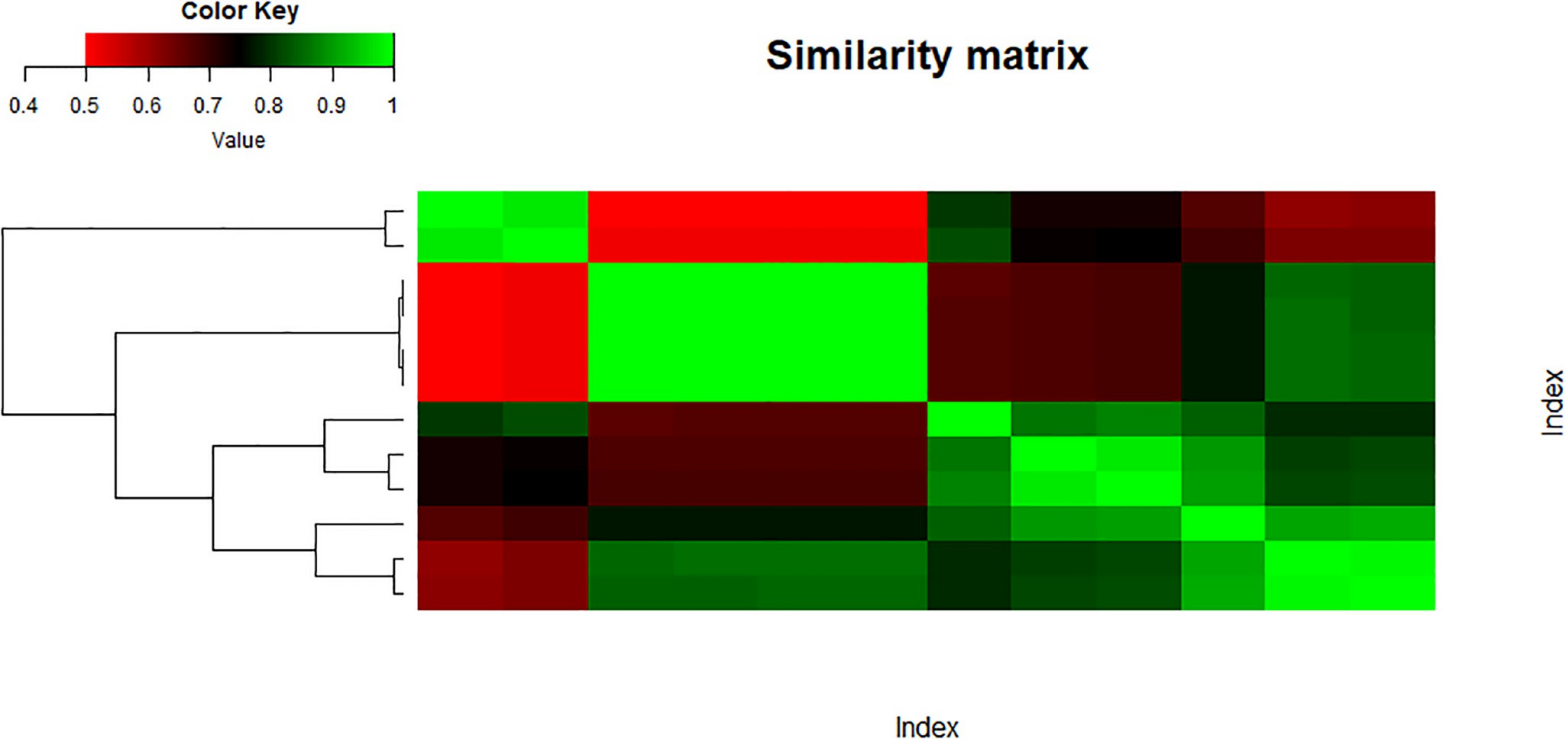
Similarity entropy matrix.

**Fig 16:**
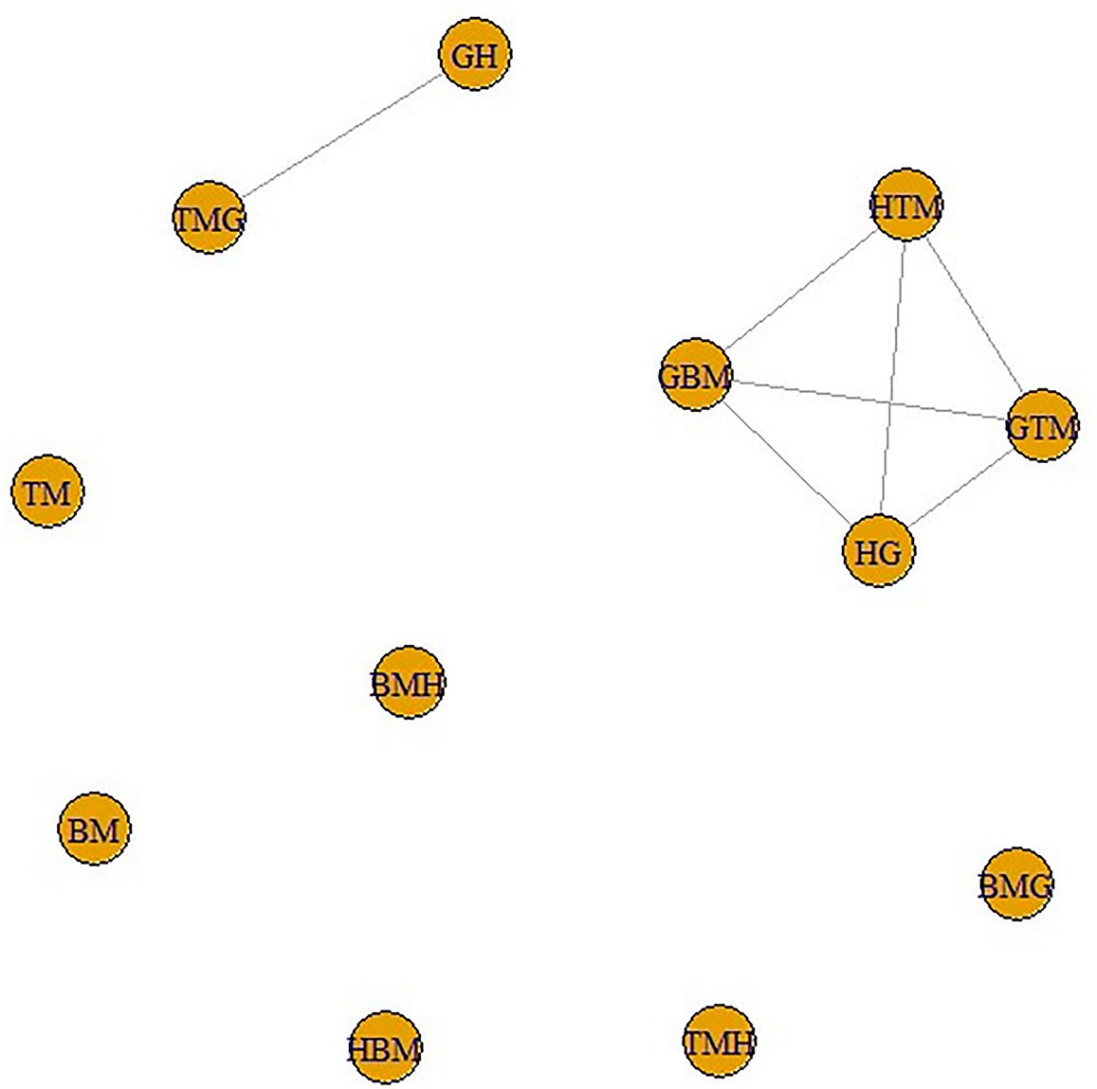
Adjacency entropy matrix.

Figs 15 and 16 show the heatmap of the similarity matrix and adjacency matrix of the data set ‘Entropy’, respectively.

**Fig 17:**
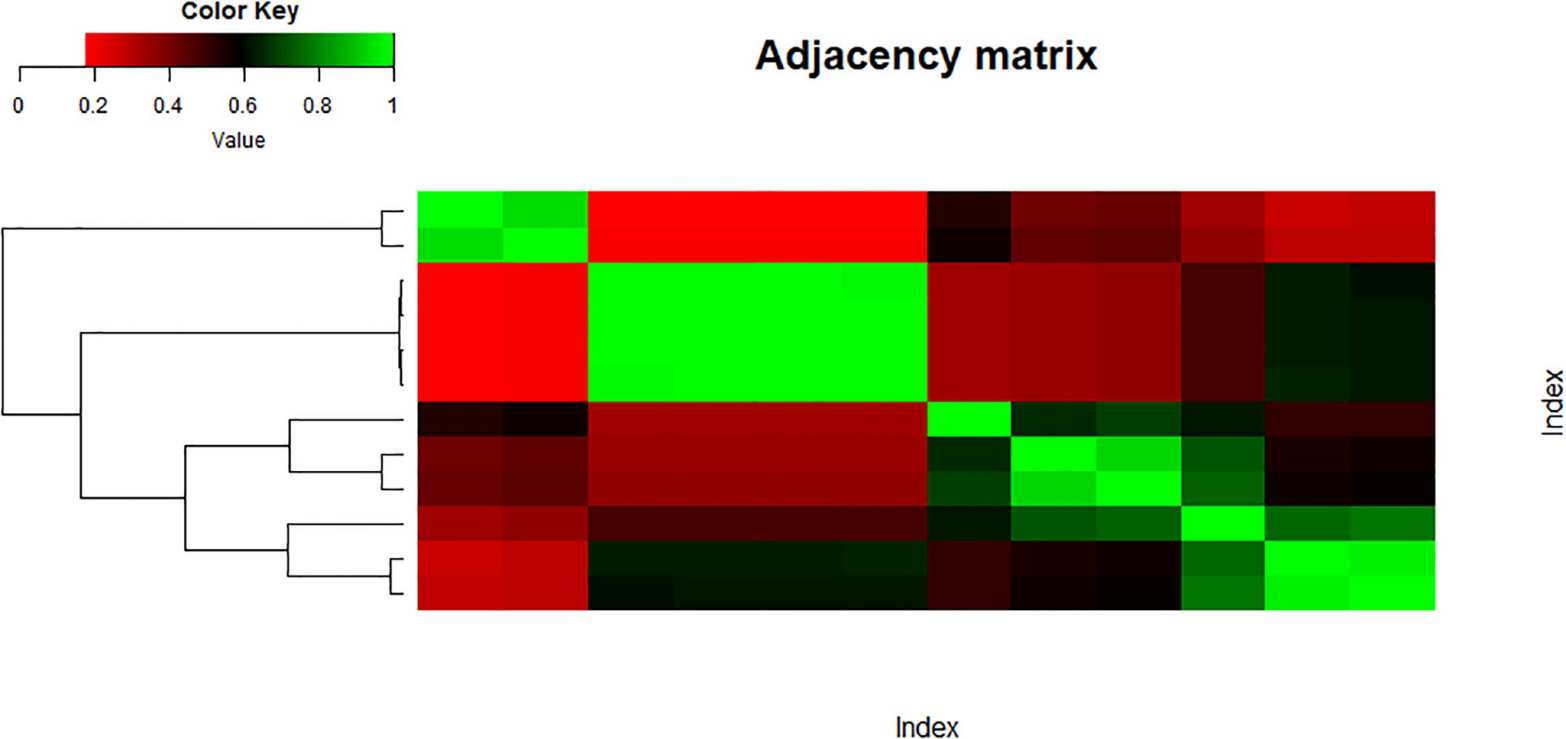
Network indices.

**Fig 18:**
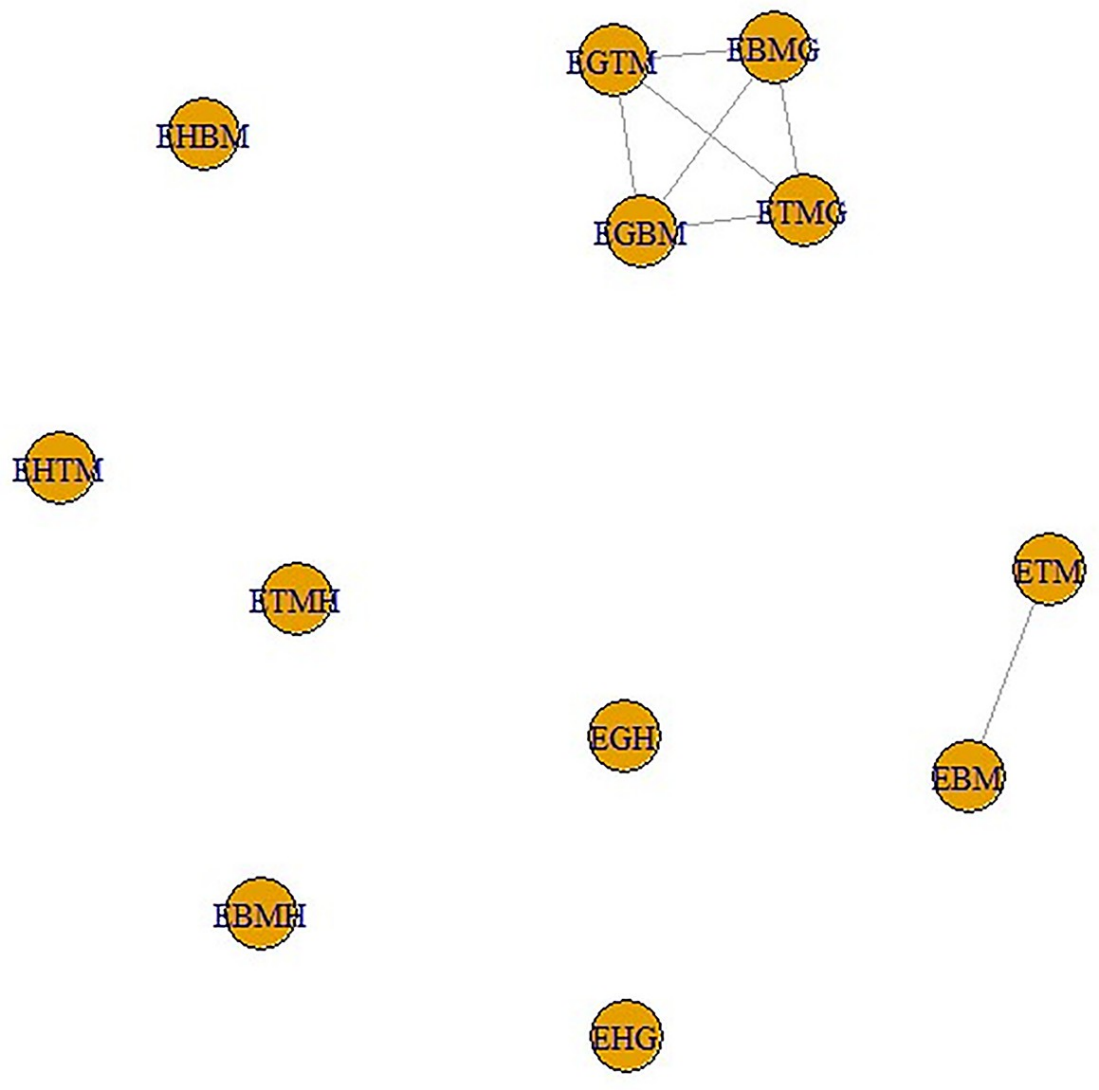
Network entropy.

Next, these adjacency matrices are utilized to infer networks. Figs 17 and 18 show the networks of data sets ‘Indices’ and ‘Entropy’, respectively.

We can easily see in both networks the structure is the same which might be considered a good sign to create mathematical models between variables of both data sets. In the next section, such models have been developed by taking variables in the data set ‘Indices’ as independent variables whereas the variables in the data set in ‘Entropy’ as dependent variables.

## 5 The logarithmic regression model

We utilized logarithmic regression analysis on our dataset to scrutinize the link between the dependent and one or more interpreter variables. This method, known as logarithmic regression, is a nonlinear regression technique in which logarithmic functions are applied to either the predictor variables or the dependent variable. We employed logarithmic transformations to identify and model any complicated, nonlinear associations in the data. Using logarithmic regression approaches, we obtained crucial insights into essential developments and configurations that would have been difficult to identify using typical linear regression techniques. A logarithmic regression model, represented by the following equation, is an effective tool for discovering and interpreting detailed correlations in the dataset:

$$\begin{array}{c} Y=a\;\text{log}\;X+b \end{array}$$

Here, Y stands for the dependent variable, X for one or more interpreter variables, *a* and *b* are estimated coefficients, and log stands for the natural logarithm function. This equation captures the essence of logarithmic regression, allowing us to model and investigate complicated nonlinear dependencies in our data.

The logarithmic models and associated statistical parameters are presented in Tables 4–7.

**Table 4:** Model for regression using logarithmic functions.

*Model*	*R*	*R* ^2^	*S*_*E*_	*SS*	*df*	*F*
*ENT*_*BM*_ = 0.954 log(*BM*) − 2.266	1	1	0.018	14.221	1	45674.266
*ENT*_*TM*_ = 0.955 log(*TM*) − 2.833	1	1	0.016	14.189	1	58453.766
*ENT*_*GH*_ = 0.950 log(*GH*) − 1.742	1	1	0.019	14.413	1	41426.547

**Table 5:** Model for regression using logarithmic functions.

*Model*	*R*	*R* ^2^	*S*_*E*_	*SS*	*df*	*F*
*ENT*_*GBM*_ = 1.023 log(*GBM*) + 1.479	1	1	0.007	13.882	1	249949.8
*ENT*_*GTM*_ = 1.019 log(*GTM*) + 2.094	1	1	0.003	13.871	1	1358532
*ENT*_*HG*_ = 1.147 log(*HG*) + 1.437	1	0.9999	0.040	14.306	1	8744.978

**Table 6:** Model for regression using logarithmic functions.

*Model*	*R*	*R* ^2^	*S*_*E*_	*SS*	*df*	*F*
*ENT*_*HBM*_ = 1.191 log(*HBM*) + 3.090	1	0.999	0.046	14.447	1	6929.241
*ENT*_*HTM*_ = 1.183 log(*HTM*) + 3.808	1	0.999	0.041	14.405	1	8396.669
*ENT*_*BMG*_ = 0.982 log(*BMG*) − 1.449	1	1	0.006	13.882	1	353790.7

**Table 7:** Model for regression using logarithmic functions.

*Model*	*R*	*R* ^2^	*S*_*E*_	*SS*	*df*	*F*
*ENT*_*BMH*_ = 0.949 log(*BMH*) − 3.251	1	1	0.018	14.654	1	47110.880
*ENT*_*TMG*_ = 0.984 log(*TMG*) − 2.046	1	1	0.003	13.874	1	1443293
*ENT*_*TMH*_ = 0.949 log(*TMH*) − 3.808	1	1	0.017	14.614	1	52862.679

A correlation coefficient (*R*) of one indicates a perfect linear relationship between the variables. When the independent variable(s) are fully responsible for all variations in the dependent variable, the coefficient of determination (*R* − *squared*) is likewise equal to 1. The standard error (*SE*) shows how well the fitted regression line matches the data points. Furthermore, the strong *F* − *statistic* emphasizes the regression model’s statistical relevance. A higher *F* − *statistic* implies that the model’s independent variables work together more effectively to explain fluctuations in the dependent variable. To put it another way, a model with a higher F-statistic fits the data better than one with a lower *F* − *statistic*. The notions of Degrees of Freedom (*df*) and Sum of Squares (*SS*) are critical to comprehending the model’s components. The sum of Squares (*SS*) is the percentage of the dependent variable’s variability that can be explained by one or more independent variables in the model. It determines how closely the data matches the logarithmic function. Degrees of Freedom (*df*), on the other hand, relate to the number of adjustable values considered in the final calculation of a statistic. It denotes the level of complexity of the model. The metrics *SS* and *df* are used to evaluate the goodness of fit of a logarithmic regression model. These metrics provide information about the model’s complexity and how well it compensates for fluctuations in the dependent variable. The graphical comparisions show the goodness of fit visually, with observed and logarithmic values forming well-fitting curves.

**Fig 19:**
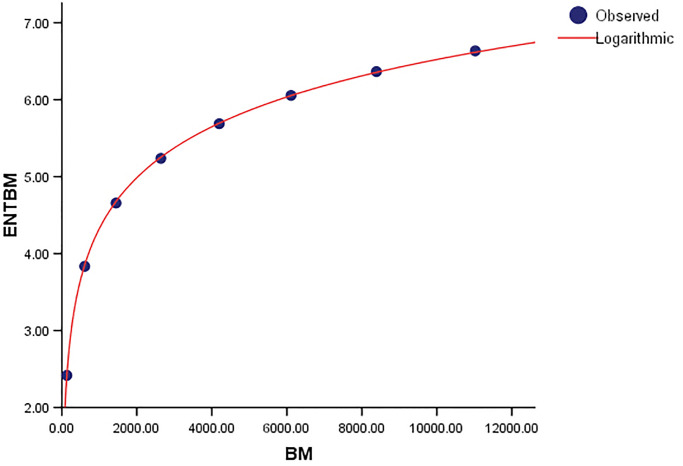
Graphical representation of the behavior of logarithmic regression (BM VS ENTBM).

**Fig 20:**
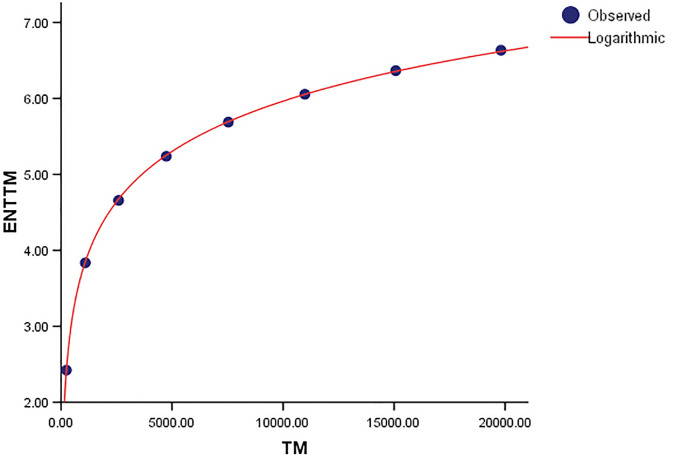
Graphical representation of the behavior of logarithmic regression (TM VS ENTTM).

**Fig 21:**
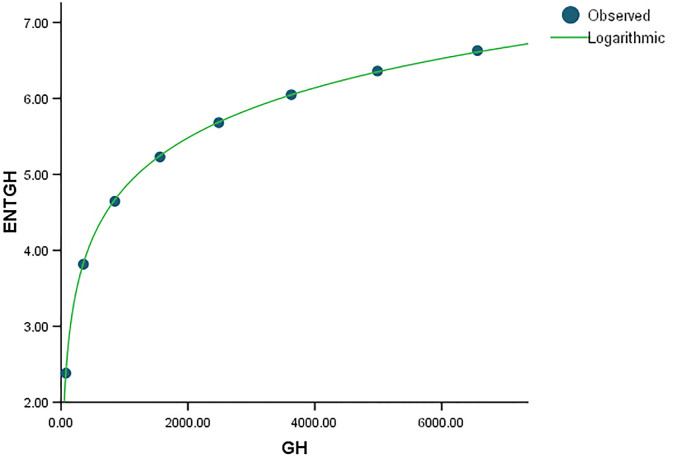
Graphical representation of the behavior of logarithmic regression (GH VS ENTGH).

**Fig 22:**
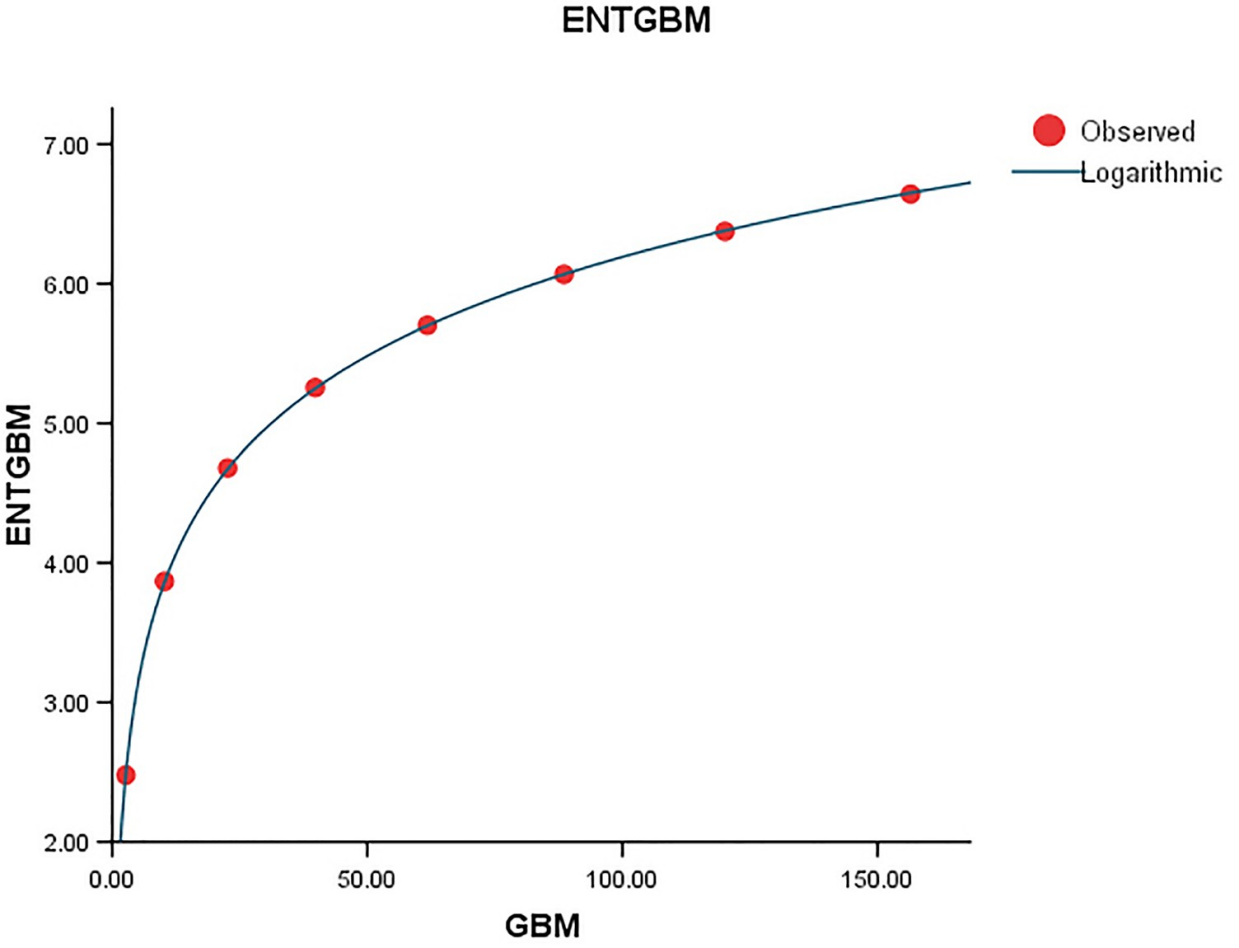
Graphical representation of the behavior of logarithmic regression (GBM VS ENTGBM).

When compared to *ENT*_*BM*_ and *ENT*_*GH*_, the *ENT*_*TM*_ F-value is much higher, indicating a significant overall relevance of the model in explaining the variance in the dependent variable, as seen in Table 4. *R* and *R*^2^ have same values. We can observe that the data points in Figs 19–22 approximately fit the curve. This could imply that the model is well-fitting.

**Fig 23:**
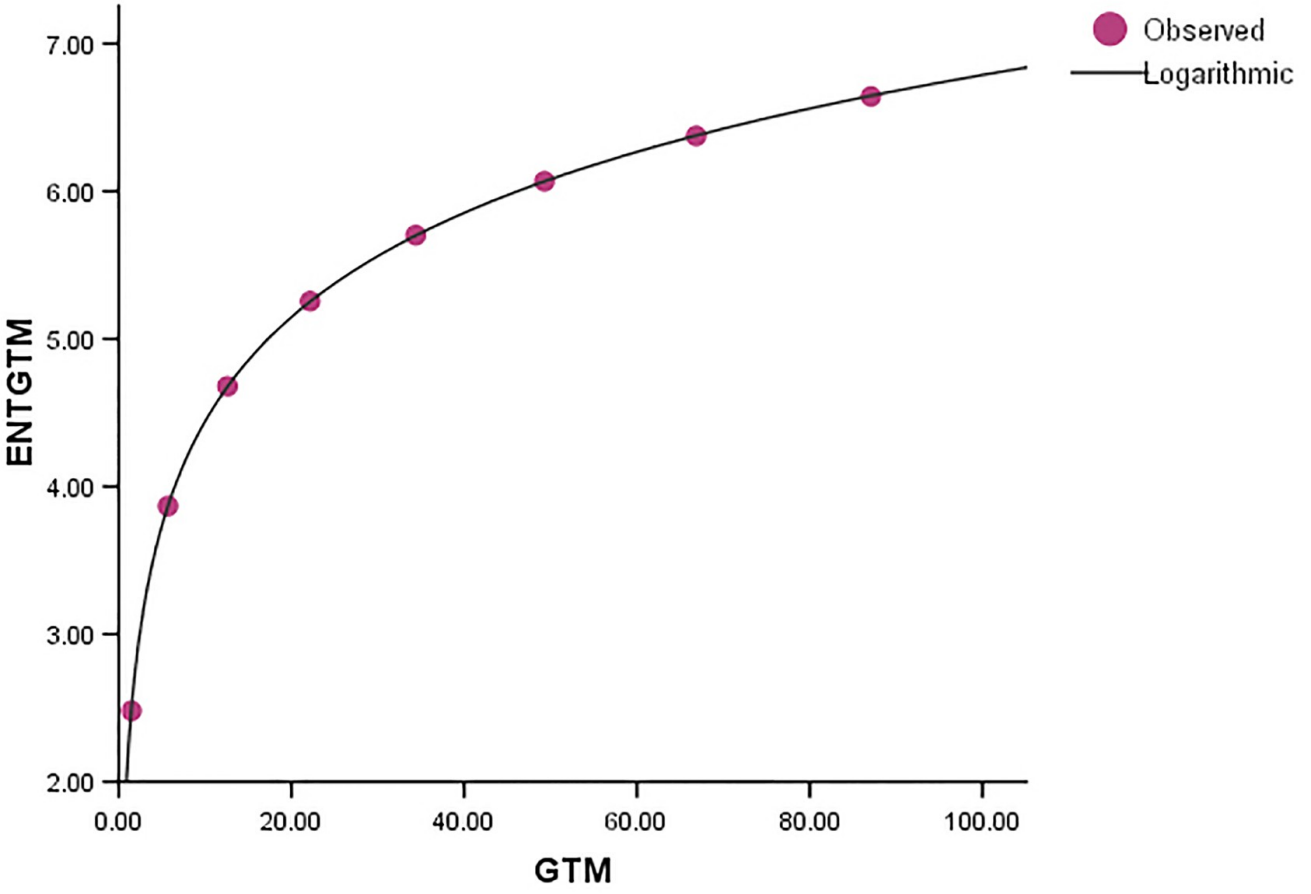
Graphical representation of the behavior of logarithmic regression (GTM VS ENTGTM).

**Fig 24:**
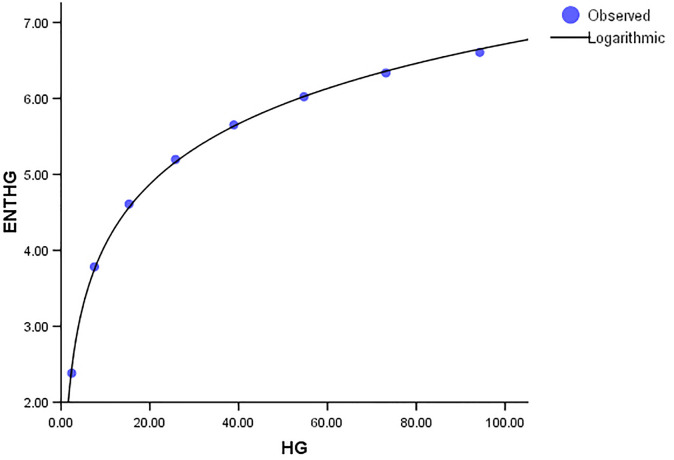
Graphical representation of the behavior of logarithmic regression (HG VS ENTHG).

**Fig 25:**
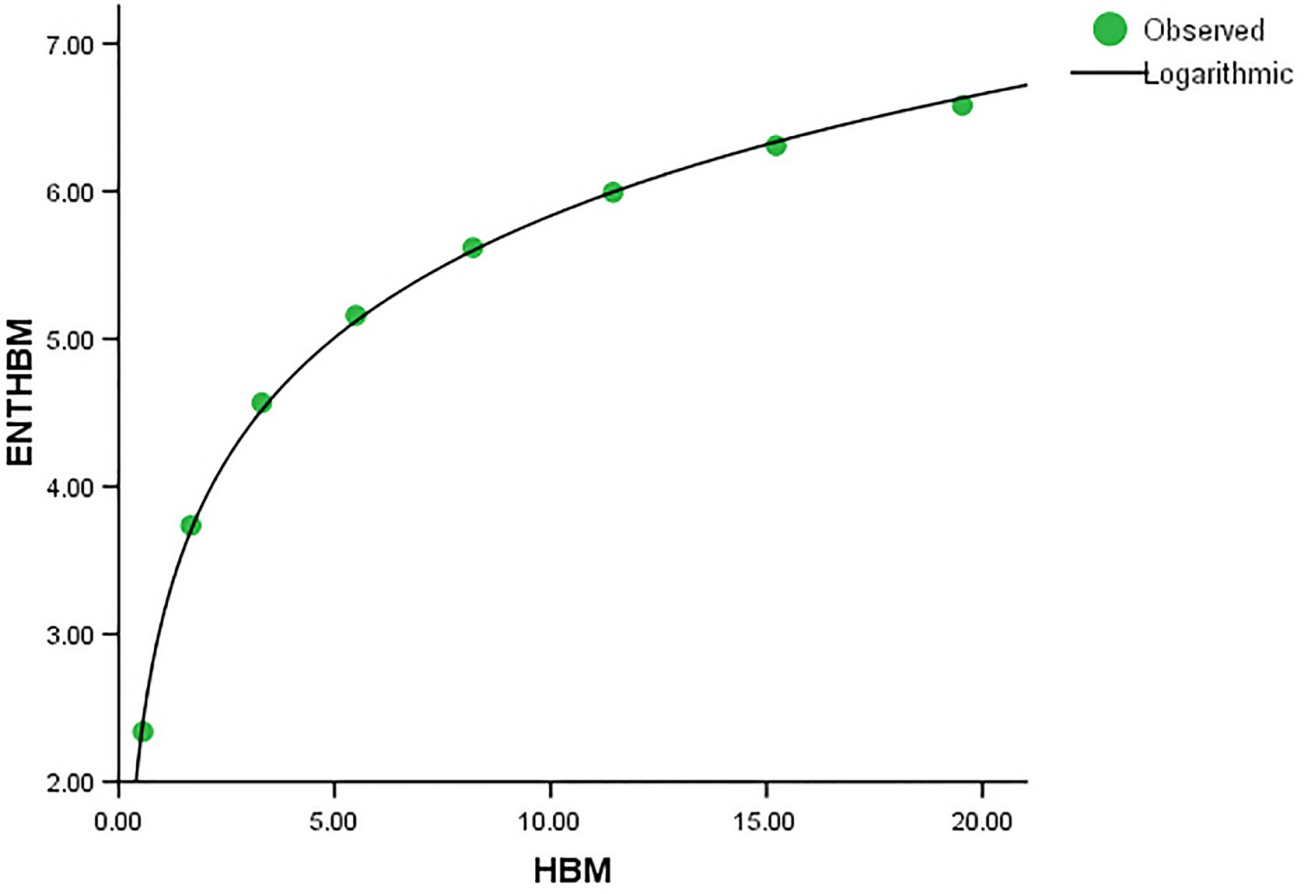
Graphical representation of the behavior of logarithmic regression (HBM VS ENTHBM).

**Fig 26:**
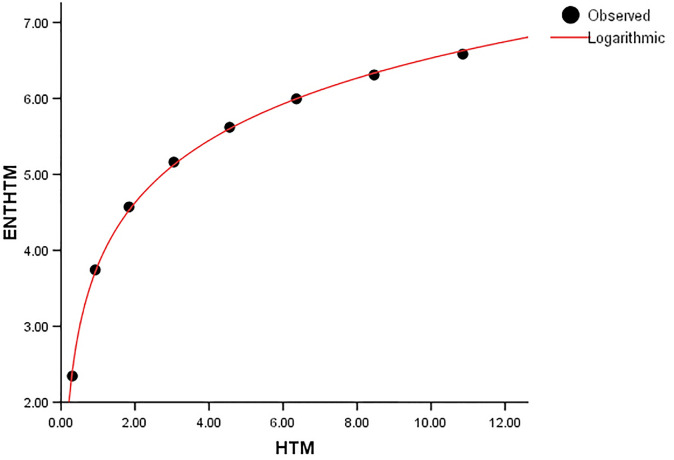
Graphical representation of the behavior of logarithmic regression (HTM VS ENTHTM).

Comparing the *ENT*_*GTM*_ to *ENT*_*GBM*_ and *ENT*_*HG*_, the F-value for the *ENT*_*GTM*_ regression model is noticeably higher, indicating a significant overall relevance of the model in explaining the variance in the dependent variable, as shown in Table 5. In *ENT*_*HG*_, *R* and *R*^2^ have slightly different values from each other, but they are identical in *ENT*_*GBM*_ and *ENT*_*GTM*_. When compared to the graphical representation in Figs 23–26 the data points closely coincide with the curve. According to this, the model appears to have a good fit by avoiding the issue of overfitting.

**Fig 27:**
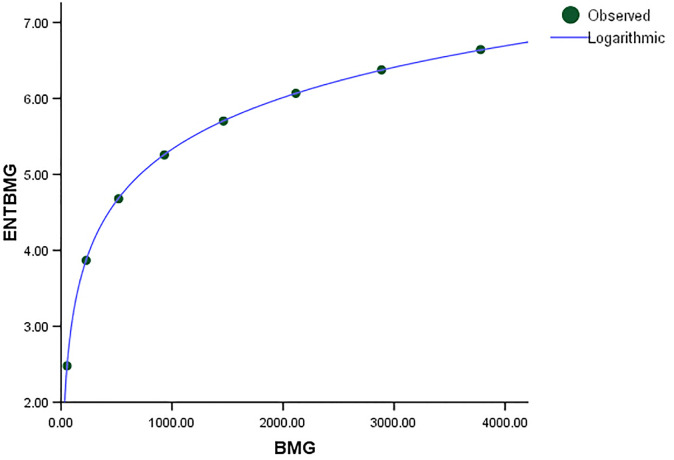
Graphical representation of the behavior of logarithmic regression (BMG VS ENTBMG).

**Fig 28:**
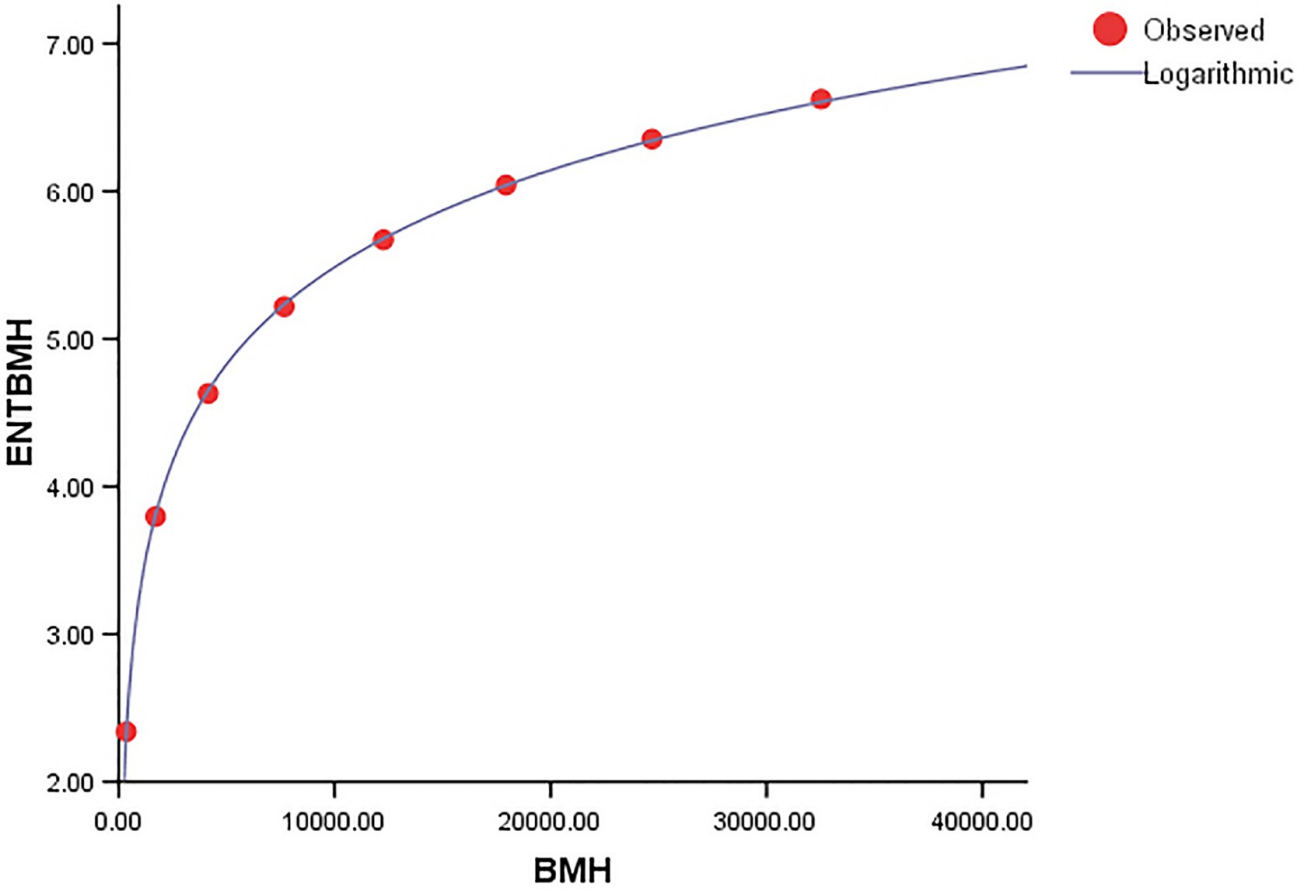
Graphical representation of the behavior of logarithmic regression (BMH VS ENTBMH).

**Fig 29:**
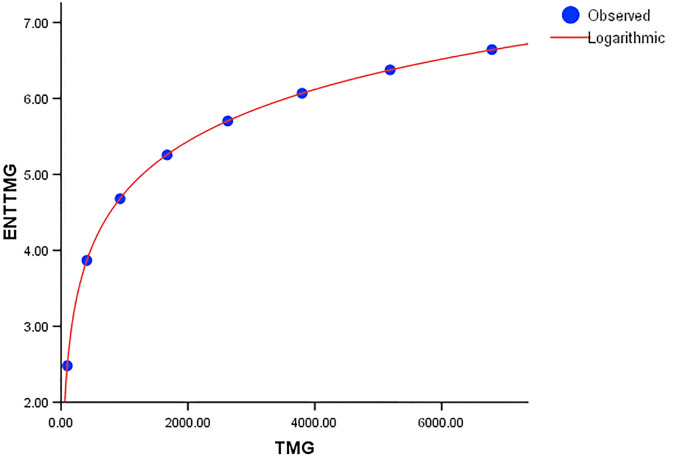
Graphical representation of the behavior of logarithmic regression (TMG VS ENTTMG).

**Fig 30:**
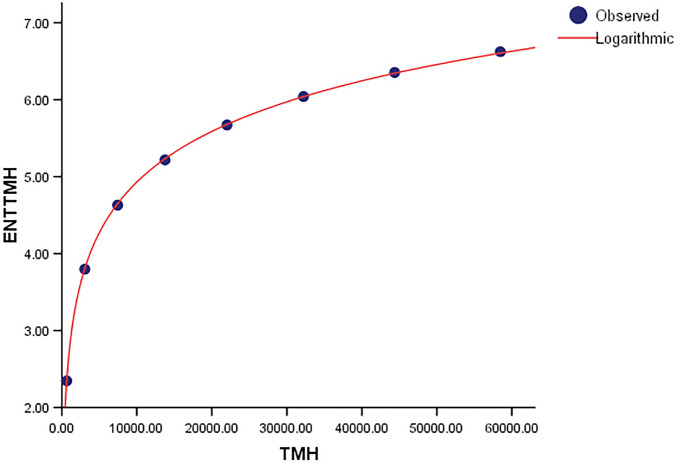
Graphical representation of the behavior of logarithmic regression (TMH VS ENTTMH).

Comparing the *ENT*_*BMG*_ regression model to *ENT*_*HBM*_ and *ENT*_*HTM*_, the F-value of *ENT*_*BMG*_ is much higher, indicating a significant overall relevance of the model in explaining the variance in the dependent variable, as shown in Table 6. Furthermore, *R* and *R*^2^ have slightly different values from each other. The graphical depiction in Figs 27–30, demonstrates that the data points closely fit the curve. This demonstrates that the model has a good fit and avoids the overfitting problem.

As shown in Table 7, the F-value for the *ENT*_*TMG*_ regression model is significantly greater than that of the *ENT*_*BMH*_ and *ENT*_*TMH*_ models, showing a strong overall significance of the model in explaining the variance in the dependent variable. Additionally, the values of *R* and *R*^2^ are the same. The graphical depiction demonstrates that the data points closely fit the curve. This demonstrates that the model has a good fit and avoids the overfitting problem.

## 6 Conclusion

In our study, we introduced two novel versions of Zagreb descriptors: Bi-Zagreb and Tri-Zagreb descriptors. Furthermore, we used a novel approach to build hybrid descriptors by merging Geometric, Harmonic, Bi-Zagreb, and Tri-Zagreb indices. These unique descriptors were thoroughly tested for their ability to predict the physicochemical properties of *CuF*_2_. We used these indices to generate entropy measures and made an important discovery: the newly developed hybrid descriptors beat their conventional equivalents. We conducted a comprehensive analysis that included logarithmic regression investigations to shed light on the interaction between these metrics and entropy. In conclusion, our research provides a significant step forward in the field of molecular descriptor analysis, with prospective applications in a wide range of chemical research and analytical sciences.
